# The spatial and temporal exploitation of anthropogenic food sources by common ravens (*Corvus corax*) in the Alps

**DOI:** 10.1186/s40462-022-00335-4

**Published:** 2022-08-25

**Authors:** Varalika Jain, Thomas Bugnyar, Susan J. Cunningham, Mario Gallego-Abenza, Matthias-Claudio Loretto, Petra Sumasgutner

**Affiliations:** 1 FitzPatrick Institute of African Ornithology, DSI-NRF Centre of Excellence, Private Bax X3, Rondebosch, Cape Town, 7701 South Africa; 2 Core Facility for Behaviour and Cognition, Konrad Lorenz Research Centre, University of Vienna, Fischerau 13, 4645 Grünau im Almtal, Austria; 3 Department of Behavioural and Cognitive Biology, University of Vienna, Djerasi Platz 1, 1030 Vienna, Austria; 4 Department of Zoology, Stockholm University, Stockholm, Sweden; 5 Ecosystem Dynamics and Forest Management Group, TUM School of Life Sciences, Technical University of Munich, Hans-Carl-von-Carlowitz-Platz 2, 85354 Freising, Germany; 6 Berchtesgaden National Park, Doktorberg 6, 83471 Berchtesgaden, Germany

**Keywords:** Non-breeder, Corvids, Space use, GPS-telemetry, Movement ecology, Displacement, Foraging

## Abstract

**Background:**

Anthropogenic food sources (AFSs) are widespread in human-transformed landscapes and the current scale at which they occur drives ecological change at the individual, population, and community levels. AFSs are exploited extensively by common ravens, *Corvus corax*. Understanding how raven populations use AFSs can provide insight into their ecological responses to AFSs.

**Methods:**

We equipped 81 ravens in the Austrian Alps with GPS-transmitters over a period of 2.75 years. Using these tracking data, we investigated how cohort differences (i.e., age, sex, and origin) and seasonal changes influence raven movement patterns (i.e., occurrence distribution and maximum daily displacement) and AFS-use (i.e., number of AFSs visited and probability of being present at any AFS) at 45 extensively exploited sites.

**Results:**

We found that proxies for experience and dominance, inferred by age (i.e., juvenile versus adult) and origin (i.e., wild-caught versus captive-bred-released) cohorts, influenced movement patterns and the number of AFSs visited. However, all individuals were equally likely to be present at AFSs, highlighting the importance of AFSs for non-breeders in the study population. Seasonal changes in environmental conditions that affect energetic demands, the availability of natural and anthropogenic food, and foraging competition,  influenced individuals’ occurrence distributions and AFS-use. We found that under harsher conditions in autumn and winter, individuals ranged wider and depended on AFSs to a larger degree. However, contrary to expectation, they were less likely to be present at AFSs in these seasons compared to spring and summer, suggesting a trade-off between time spent moving and exploiting resources. We attribute the small ranging movements exhibited by non-breeders in spring and summer to the presence of highly territorial and socially dominant breeders. As breeders mostly stay and forage within their territories during these seasons, competition at AFSs decrease, thereby increasing the likelihood of individuals being present at any AFS.

**Conclusions:**

We emphasize that movement and AFS-use differ according to cohort differences and the seasonality of the environment. Our results highlight that predictable AFSs affect foraging strategies among non-breeding ravens. The extent of AFS-exploitation among non-breeding ravens in our study emphasize the potential of AFSs in shaping raven movement and resource-use.

**Supplementary Information:**

The online version contains supplementary material available at 10.1186/s40462-022-00335-4.

## Background

Humans have supplied food deliberately and unintentionally to wild animals for over 10,000 years, however, the current scale at which anthropogenic food sources (AFSs) occur is driving ecological change [1–3]. At the individual-level, animals exploiting AFSs can experience changes in fitness (e.g., survival, reproduction) and behaviour (e.g., dispersal, foraging) [4–8]. These individual-level effects can trigger cascading changes in populations and communities (e.g., population density, trophic-level interactions), and impact overall ecosystem functioning [1, 2, 9]. It is increasingly important to understand the multiple layers of responses shaped by AFSs, given the growing and dynamic human footprint [10]. Especially under scenarios of changing AFS availability, knowledge of how populations utilise AFSs can provide insight into their ecological responses.

Anthropogenic food sources alter the quantity and quality of available resources in the environment, creating novel ecological conditions often characterized by high environmental predictability. AFSs are spatially concentrated, abundant, and stable in both space and time compared to natural food sources that are often sparsely distributed and ephemerally present [1, 11, 12]. Additionally, AFS availability often follows scheduled patterns in human activity, for example waste centres or schools operating on certain daily (7:00 am to 5:00 pm) and weekly (Monday to Friday) cycles [13, 14]. These characteristics of AFSs increase environmental predictability and influence the costs and benefits of animal foraging [11, 15]. According to optimal foraging theory, individuals should forage at AFSs if it is energetically advantageous to do so [16]. Animals may therefore exhibit different individual foraging strategies to benefit from AFSs compared to conditions with limited human influence, which could include changes in resource tracking and preferences [13, 17], time spent foraging [18], and food-searching efforts [19].

The common raven, *Corvus corax*, is an ecological generalist and synanthrope that thrives in human-transformed landscapes, extensively exploiting AFSs and surrounding areas [20–23]. Like other corvid species, ravens are socially and cognitively complex, and behaviourally and ecologically flexible. With these traits, they are adaptable to the dynamic conditions of anthropized areas (e.g., to human interference in both infrastructure and disturbance), and can benefit from the foraging opportunities available [3, 24, 25]. Consequently, several corvid populations across the globe have been expanding, likely aided by climate change, land-use change, and relaxation of persecution pressure [26, 27]. For raven populations, AFS exploitation has often resulted in increasing density and wider distribution through increases in individual reproduction [25] but also see [28], adult and offspring survival [7, 29], and decreases in winter mortality [30–32]. In Central Europe, where ravens were almost extinct in the mid-1900s, population growth represents recovery [27, 33, 34]. However, studies from North America highlight that population growth also has the potential to raise ecological and conservation issues including increased predation on threatened species and human-wildlife conflict [7, 25, 35].

The extent to which ravens exploit AFSs can shape ecological implications for conspecifics and other species. Among raven populations, non-breeding individuals use AFSs differently to territorial, breeding individuals. Non-breeders can travel farther than their breeder conspecifics to exploit AFSs [36] and with large numbers of non-breeders at an AFS, neighbouring breeding pairs can fail to monopolize the food source [28]. With the capacity for widespread movements, non-breeders raise concerns regarding spill-over predation, whereby individuals subsidized by AFSs invade adjacent areas, inflating predation and impacting trophic networks there [37, 38]. Far-ranging flights also pose a challenge for the non-lethal conservation management of problematic non-breeder populations, such as translocations [39] and the control of food sources [25]. Addressing these issues are complex as within non-breeder groups, individuals can differ widely in terms of foraging preferences and space use [33, 40, 41], based on age and origin (e.g., wild-reared versus released from captivity) cohorts [33, 42] and on external factors (e.g., resource type, seasonality of the environment) [23, 31]. However, by investigating individual movement patterns, we can better understand the nuances in foraging strategies and decisions made by individuals within a population [4–6, 24, 43].

In this study, we used long-term GPS-tracking data from non-breeding ravens in the Austrian Alps to investigate how differences between individuals, cohorts and external factors influence space and AFS-use. For each season and year that individuals were tracked in, we analysed their occurrence distribution and maximum daily displacement to understand movement patterns. The occurrence distribution reflects the extent of the landscape used and potentially accessible to individuals (i.e., how vagrant birds are), and the maximum daily displacement provides an understanding of which individuals pursue long- versus short-range movements [44, 45]. For AFS-use, we first identified extensively exploited AFSs in the landscape. We then quantified the number of AFSs visited by individuals and their probability of being at any AFS in each season and year. Based on previous studies, we predicted that movement and AFS-use would vary widely among individuals and cohorts. For the latter, we expected differences between age (i.e., juvenile versus adult) and origin (i.e., wild-reared versus captive-released) cohorts, but not sex [23, 42, 46, 47], as both categories reflect potential differences in experience and social ranking [33, 48]. We also expected seasonal differences, with wider-ranging movement patterns and increased AFS-use in winter as natural resources in the environment deplete, thermoregulatory expenses increase, and some predictable anthropogenic seasonal food sources such as ski huts become available [23, 32, 49]. Using movement data to characterize foraging behaviour, we highlight how non-breeding ravens in a semi-transformed alpine landscape use AFSs and discuss the potential effects of AFSs on movement and resource-use.

## Methods

### Study site, species and GPS tracking

The Eastern Alps in Austria have a high elevational gradient reaching up to approximately 3,800 m above sea level (a. s. l.). The vegetation is characterized by broad-leaved forests up to 600 m a. s. l. that transitions to conifer-dominated forest above 1,200 m a. s. l. and to alpine vegetation above the tree line, which ranges between 1,800 and 2,200 m a. s. l. Mean annual precipitation and temperature vary greatly with elevation and location, ranging from 600 to 2,500 mm and − 5 to 11 °C respectively [50]. Land-use at lower elevations is dominated by forestry, mining, tourism, hunting, and recreation. In this region, non-breeding ravens aggregate in large numbers at AFSs including game parks, compost sites, and garbage dumps, which provide food throughout the year, as well as farms, ski huts, restaurants, and hotels, which operate more seasonally (with the latter three peaking in food supply across winter months) [40]. Naturally occurring food resources include carcasses, small vertebrates, grains, insects, and fruits.

**Fig. 1:**
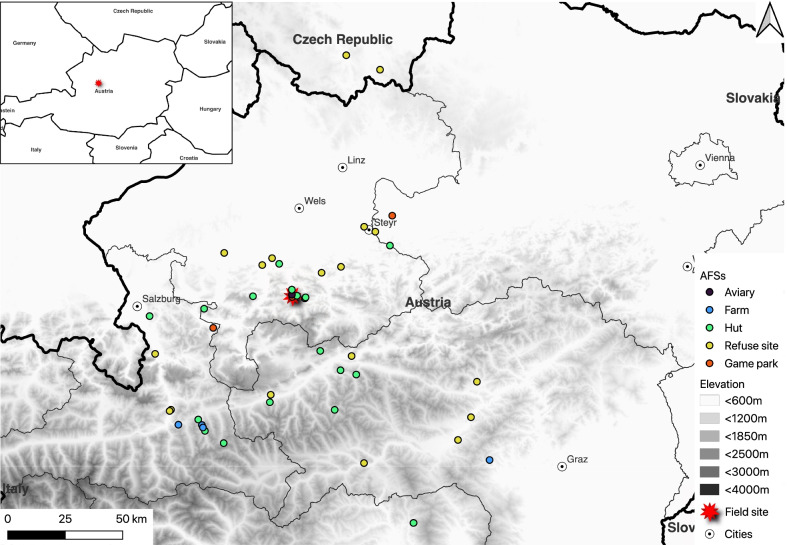
The field site, where ravens were tagged with GPS-transmitters, is situated at the northern edge of the Eastern Alps in Austria, indicated with a red star. The location of 45 anthropogenic food sources that were identified in this area are shown as points, grouped by resource type, against the background elevation. Major cities are indicated as white and black circles. The box in the upper left corner shows the field site in Austria and the countries neighbouring Austria. Thick black lines show the country borders while thin black lines depict country states

We tagged ravens with GPS-transmitters at our field site in the inner Alm valley, which is situated at the northern edge of the Eastern Alps in Austria (Fig. 1). Wild-caught individuals were captured using drop-in traps [51] baited with meat and bread, placed in Cumberland Wildpark (47°48′19.08″ N, 13°56′55.32″ E). At this game park, ravens scrounge food from captive animals all year long, reaching numbers of approximately 30 individuals in summer and up to 120 in winter [14, 23]. The frequent use of the game park by ravens has made it a reliable trapping location and facilitated the long-term monitoring of the local population, with over 500 individuals marked with patagial wing tags and / or coloured leg-rings since 2007 [48]. At the Konrad Lorenz Research Station (KLF), situated 700 m away from the game park, some raven individuals that were parent-raised in captivity were released into free-flight (80 m^2^) [33]. Since 2018, the release of captive-bred juvenile ravens has been part of a research program on early life experiences, and once released approximately 6 months after hatching, GPS-tracking provides a way to understand how individuals inexperienced with the surrounding landscape adapt to available resources. Captive-released individuals generally integrate successfully into the non-breeding local population within a month or two [33].

All study individuals were fitted with backpack-style, solar-powered GPS-transmitters (OrniTrack-25 with elevated solar panels, Ornitela UAB, Lithuania; https://www.ornitela.com/25g-transmitter). Similar to GPS-transmitters described and used in Loretto, Schuster [23], these weighed 25 g (27 g including the attachment with Teflon harness and aluminium crimps) and never exceeded 3% of the bird’s bodyweight. In the Alm valley population, the average weight of females (n = 213) is 1025 ± 70 g SD and males (n = 256) is 1150 g ± 120 g SD. Sex was determined genetically from blood samples collected when individuals were trapped. Data from the transmitters are downloaded via GSM/GPRS/3G network, stored, and managed on Movebank, a database for animal tracking data. The datasets generated and analyzed in this study are available in the Movebank Data Repository (10.5441/001/1.22nd28v7).

The non-breeder demographic comprises sexually immature juveniles (within their first year) and sub-adults (2nd and 3rd year), and sexually mature adults (3rd year onwards) that lack either a partner or defendable breeding territory [40]. Within this population, some adults can take 10 years or more to become a breeder (Loretto & Bugnyar unpublished data). Breeders maintain large territory sizes of 10km^2^ or more [52], which results in limited area available for other raven pairs. With a life expectancy of 10 to 15 years in the wild, ravens spend a considerable amount of time as non-breeders, as opposed to territorial breeders [42, 53]. We assigned age class based on the coloration of the mouth lining (pink for juveniles, black for sub/adults) and plumage (slightly brown for juveniles, black for sub/adults) [23], and age classes reflect the estimated minimum age. Although sexual maturity occurs at around three years of age [46], we combined the sub-adult and adult categories (> 1 year old; hereafter referred to as the ‘adult’ age class) as our study individuals were exclusively non-breeders (i.e., visual inspection of movement during breeding season did not reveal nesting activity or territorial behaviour) and we could not determine the exact age of adults at trapping (i.e., only age estimates). Furthermore, being their first year of life, juveniles have limited experience with the foraging context compared to older birds that have survived one or more winters - the season in which juveniles are most likely to die [7]. Thus, in grouping sub-adult and adults, we account for experiential age class differences.

### Data analysis

We used movement data from a total of 81 non-breeding ravens, tracked between July 2017 and March 2020, of which 13 were tracked since 2017, 39 since 2018, and 29 since 2019. There were 56 wild-caught and 25 captive bred-released individuals, and 49 females and 32 males. At the time of trapping, 51 individuals were tagged as juveniles (including the 25 captive bred-released birds), 20 as sub-adults, and 10 as adults. As individuals hatch in spring, the start of spring across all years (i.e., March 21) marked when an individual transitioned between juvenile and adult age classes in our analyses (Additional file 1).

We divided the data according to astronomical seasons using the equinox dates of March 21, June 21, September 23 and December 21 as they reflect changes in food resource distribution and availability [23]. These dates also roughly correspond to seasonal changes in raven behaviour [40]. Nest building and breeding occurs between February and March (i.e., winter); chicks hatch in spring and fledge towards the end of May; fledglings stay with their parents in summer and integrate in non-breeder flocks in autumn. As tracking occurred over different lengths of time, the number of individuals for which GPS-tracking data were available in each season and year (hereafter referred to as ‘individual-season-year combinations’) varied across the study duration (Additional file 1). From our sample, tracking ended for various reasons including 23 confirmed and suspected mortalities and 13 equipment failures (i.e., GPS transmission/battery failure and GPS backpack loss). All metrics were estimated and analyses were conducted using the statistical software R (version 4.1.1) [54].

### Movement

For each individual-season-year combination, we estimated the occurrence distribution as the 95% utilisation distribution (UD) (Additional file 1) with dynamic Brownian Bridge Movement Models (dBBMM) from the “move” package [55]. A utilisation distribution describes the probability of where an animal occurs in an area at any randomly chosen moment [56] and the dBBMM approach builds on previous UD estimations by considering both the sequence of fixes along a trajectory and the time between fixes. It is particularly useful in estimating the UD for data with non-regularly sampled tracks, accounting for temporal autocorrelation and high data volumes [57]. Data were collected from sunrise to sunset, with one GPS-fix collected 6 h after sunset. Given the seasonality of daylight length in northern temperate latitudes, the ratio of daytime to night-time GPS fixes was higher in months with a longer daylight duration compared to months with a shorter daylight duration. To focus on daytime foraging, roosting and social activity at AFSs consistently across the year, we filtered the data to retain only daytime GPS-fixes, between sunrise and the start of sunset [58]. The data are of varying sampling frequency as collection depended on the available energy of the solar powered batteries in the tracking units and the objectives of other investigations on the data. The sampling ranged from every 1 s to 5 min in summer to every 1–2 h in winter, with occasionally even larger time lags.

To estimate the dBBMMs, we used the following parameters: margin of 13, window size of 31, an estimated location error of 20 m, raster cell size of 100 m, “timestep” as 1 min, and for calculating the variance, we excluded time lags that exceeded 5 h. We also measured the maximum daily displacement as the distance between an individual’s furthest GPS fix relative to its first GPS fix within a day, averaged per individual-season-year combination (Additional file 1). In addition to the UD, this metric provides an understanding of movement independent of the sampling rate.

### AFS-use

#### Identification of AFSs

To identify yearly and seasonal AFSs, we plotted the tracking data to find clusters of GPS-fixes (i.e., at least 10 GPS fixes within a radius of 200 m) that were in proximity to anthropogenic features using high-resolution satellite imagery on Google Earth Pro (version 7.3.4.8248) (available online at http://www.google.com/earth/index.html). Given the irregularity in sampling rates across seasons (i.e., lots of GPS-fixes and clusters in summer versus winter) we only plotted GPS-fixes at a minimum of 15-min intervals. We extracted the GPS coordinates (taken as the centre point of a single or group of clusters) for a total of 47 sites.

From these 47 sites, we were able to clearly identify 21 large and long-term AFSs on Google Earth including 11 compost sites, 7 waste-management centers and 3 game parks (Additional file 2). From the remaining 26 sites that were smaller-scale and seasonal, we ground-truthed 11 sites with the assistance of a field technician. We were able to confirm that 9 of the 11 were indeed AFSs. The other two sites were found to be roosting or socializing areas near buildings (i.e., trees near farmhouses) with no obvious food supply and were excluded from the analysis. For the 15 AFSs that we did not ground-truth, we used the company names and profiles at the respective coordinates on Google Earth to deduce information about the foraging sites. If the misidentification rate of 18.2% (i.e., 2 out of 11 sites) from the ground-truthing also applies to these 15 remotely identified sites, then approximately 3 of them could potentially be roosting areas instead of AFSs. With two AFSs excluded from the original 26 small-scale sites, we were left with 24 smaller-scale and seasonal sites. These included 5 farms, 18 ski huts, hotels and restaurants, and the KLF aviary, where food continued to be provisioned for a few weeks after the captive-bred individuals had been released.

Taken together, the 21 larger-scale sites and 24 smaller-scale sites gave us a final sample of 45 food sources (Additional file 3; Fig. 1). The different AFSs were grouped into the broader resource types of ‘game parks’, ‘refuse sites’ and ‘huts’ (Table 1) based on similarities in site descriptions obtained from Google Earth satellite imagery (Additional file 2) and the field site visits.

**Table 1:** General description of the different anthropogenic food sources (AFSs; n = 45) used by common ravens *Corvus corax*, in the Austrian Alps and the resource types they were grouped in for the analyses based on similarities in site descriptions. Site descriptions for game parks (n = 3) and refuse sites (n = 18) were obtained from Google Earth satellite imagery (Additional file 2). Site descriptions for huts (n = 24) were obtained from field site visits (n = 9) and Google Earth (n = 15)

Resource type	AFS	Description
Game parks (n = 3)	Game parks (n = 3)	Game parks have animal enclosures (e.g., wild boars, fallow deer, wolves, bears) with daily food supply for captive animals
Refuse sites (n = 18)	Compost sites (n = 11)	Contain long, tall (~ 1.5 m) rows of household and organic waste matter, mixed with woodchips and sawdust
	Waste management centres / dumps (n = 7)	Combine both organic and non-organic material. Non-organic waste included bales and large piles of plastics
Huts (n = 24)	Ski huts, hotels, restaurants (n = 18)	Particularly active during winter tourism, many sites dispose kitchen scraps/garbage in the forest next to the main building (some illegally)
	Farms (n = 5)	Some farms were found to have small composting area close to buildings where barnyard animal waste is processed. At the end of winter, when animals are moved out to the fields, the barn compost is often raked out to compost in warming weather Other farms had offal piles and other waste available for ravens to scavenge, increasing in abundance over the hunting season
	KLF aviary (n = 1)	A spacious outdoor aviary (80m^2^) at the Konrad Lorenz Research Station where meat scraps are placed for a few weeks after the release of captive-bred individuals

#### Quantifying AFS-use

We used the ‘recurse’ package [59] to examine how the number of revisitations at each identified AFS changed with buffer sizes up to 150 m in radius, beyond which individuals were not considered to be using the site. Site-specific buffers were then selected based on the best estimate of the distance above which revisitations no longer increased with radius size, or at the maximum of 150 m. For game parks (n = 3) and huts (n = 24), optimal buffer radii had a median of 80 m (ranges: 50–100 m and 20–150 m respectively). Refuse sites (n = 18) had larger radii of median 100 m (range: 50–150 m) (Additional files 3 and 4). Buffers encompassed buildings and surrounding trees where ravens frequently aggregate to roost and socialize. Such aggregations can be of large numbers, sometimes exceeding hundreds of individuals [23, 32, 46, 60]. For each individual-season-year combination (n = 376), we counted the number of AFSs visited based on the number of unique site-buffers each individual intersected with. To determine the probability of being at any AFS, we then calculated the proportion of GPS fixes within and outside a buffer for the individual-season-year combinations where the number of AFSs visited was at least 1 (n = 369). Given all individuals were either trapped or released at AFSs (i.e., Cumberland Wildpark and the KLF), the 7 combinations reporting zero AFSs visited were likely missed due to irregular sampling, short tracking times (< 24 days) and / or the possibility that they did not use an AFS post-release. Thus, these data might not be true zeroes but are presented as such in the results.

### Modelling approach

We used generalised linear mixed models (GLMMs) with the ‘glmmadmb’ package [61, 62] and multi model inference with the ‘MuMIn’ package [63] to test which factors influence the occurrence distribution, average maximum daily displacement, the number of AFSs visited and the probability of being at any AFS. Collinearity among predictors was tested using the variance inflation factor (VIF) in the “car” package [64]. All VIFs were < 2, indicating that none of the predictors violated the assumption of independence [65].

The global model for each response variable included age class (i.e., juvenile, adult), origin (i.e., wild-caught, captive-released), season (i.e., autumn, winter, spring, summer), year and sex (i.e., male, female) as fixed factors. Year was included to account for inter-annual differences, but with only 4 factor levels available (2017, 2018, 2019 and 2020) we could not include it as a random factor [66]. To control for variation in sampling rate, we included the ratio of the number of GPS fixes to tracking days for each individual-season-year combination as a fixed factor in all models except for the model estimating the probability of being at any AFS, where the sampling rate was already accounted for (i.e., by calculating the proportion of GPS fixes within AFS buffers versus outside). Individual identity was included as a random factor in all models to account for non-independence in the data. We modelled the occurrence distribution and average maximum daily displacement with a log-normal distribution (identity-link function), the number of different AFSs with a Poisson error distribution (log-link function), and the probability of being inside any AFS with a binomial distribution (logit-link function). We checked the models for dispersion issues and included an additional observation-level random effect (ORLE) for the probability of being at any AFS to account for overdispersion in our binomial data [67].

We followed a multi-model inference approach and compared models with all possible predictor combinations of the global model to test competing hypotheses. The resulting ranked and weighted models are indicative of the relative support for each hypothesis [68, 69]. Models were ranked according to Akaike’s Information Criterion corrected for small sample sizes (AICc) and we report the model average results for the candidate models extracted above a threshold of ΔAICc ≤ 6 [70] (Additional file 5).We present the direction and magnitude of the parameter estimates, unconditional standard errors (Unc. SE), 95% confidence intervals (95% CI), and the relative variable importance (RVI) of the model parameters. Unconditional SEs incorporate model selection uncertainty compared to standard SEs which only consider variance [69]. In the text, we report the back-transformed coefficients and confidence intervals as ‘coefficient [2.5%, 97.5%]’. We also summarize the raw data as ‘mean ± 1 SE’ followed by range. A term was considered a good predictor of the response variable if it had a high RVI above 80%, and an effect was interpreted as positive or negative if CIs did not overlap zero.

## Results

Our analysis included a total of 2,889,700 GPS fixes from 81 non-breeding ravens that were tracked between July 2017 and March 2020. On average, we had 35,675 ± 6,300 (56–201,913) GPS fixes and 286 ± 20 (7–674) tracking days per individual.

### Movement

Individuals exhibited great variation in their occurrence distributions (108.22 ± 16.42 km^2^; 0.02–2,882.65 km^2^) (see Loretto, Schuster [23] for previous estimates based on 10 non-breeders from the same population) (Additional file 6) and the average maximum daily displacement (3.96 ± 0.21 km; 0.04–18.97 km) per season and year (n = 376).

**Fig. 2:**
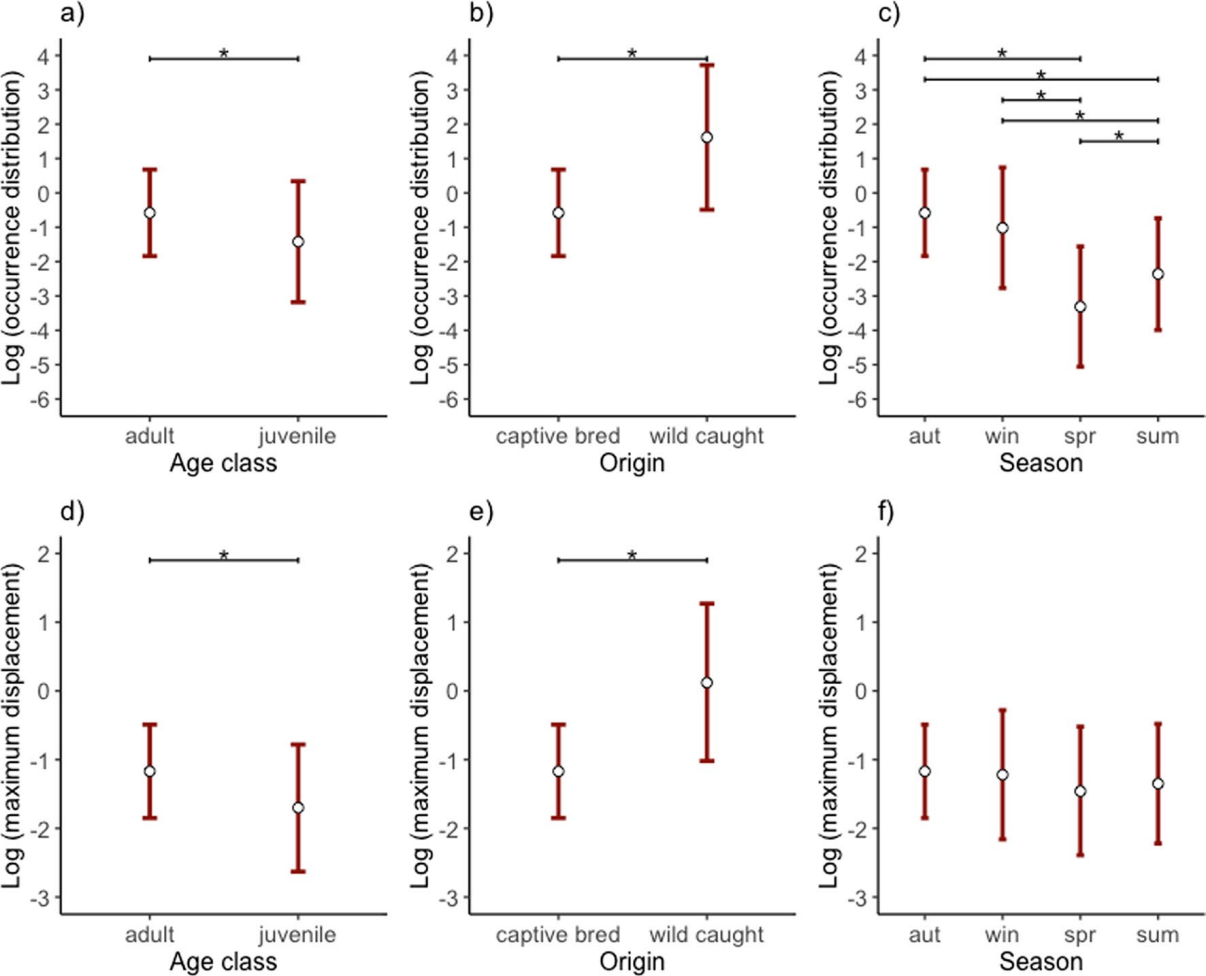
The occurrence distribution (presented on a logarithmic scale) calculated from 81 GPS-tagged non-breeding common ravens (*Corvus corax*) in the Austrian Alps differed between **a** adults and juveniles, **b** captive-released and wild-caught individuals, and **c** seasonally. The averaged maximum daily displacement (presented on a logarithmic scale) also differed between **d** adults and juveniles, **e** captive-released and wild-caught individuals, but not **f** seasonally. White circles with red error bars depict model-averaged estimates from a generalised linear mixed model (GLMM), with 95% confidence intervals (Table 2)

Age class, origin and season were the most important predictors explaining the occurrence distribution (Table 2; Fig. 2a–c). The occurrence distribution was lower for juveniles than adults by − 56.82% [− 73.75, − 28.97], and 800.34% [288.17, 1988.28] higher for wild-caught individuals than captive-released individuals. The occurrence distribution was largest in autumn followed by a decrease in winter by − 35.46% [− 60.53, 5.52], − 83.15% [− 88.29, − 75.77] in summer and − 93.50% [− 96.00, − 89.42] in spring, with differences noted in both autumn and winter to summer and spring, and between summer and spring (Additional file 7).

**Table 2:** Model averaging outputs for the top models based on ∆AICc <  = 6 for (a) the occurrence distribution (log-normal distribution) and (b) average maximum daily displacement (log-normal distribution) for 81 individual ravens GPS-tagged for 2.75 years in the Austrian Alps

**a**) Occurrence distribution (n = 376 estimates from 81 birds)							
Log-transformed model estimates					Back-transformed estimates as % change		
Fixed effects	Estimate	Unc. SE	95% CI	RVI	Estimate	Unc. SE	95% CI
*Intercept*	− 0.58	0.64	[− 1.84,0.68]		− 43.99	90.01	[− 84.15, 97.86]
Juvenile^Ϯ^	− 0.84	0.25	**[**− **1.34, **− **0.34]**	**1.00**	− 56.82	28.81	[− 73.75, − 28.97]
Wild-caught^+^	2.20	0.43	**[1.36, 3.04]**	**1.00**	800.34	53.39	[288.17, 1988.28]
Spring^‡^	− 2.73	0.25	**[**− **3.22, **− **2.25]**	**1.00**	− 93.50	28.12	[− 96.00, − 89.42]
Summer^‡^	− 1.78	0.18	**[**− **2.14, **− **1.42]**	**1.00**	− 83.15	20.30	[− 88.29, − 75.77]
Winter^‡^	− 0.44	0.25	**[**− **0.93, 0.05]**	**1.00**	− 35.46	28.40	[− 60.53, 5.52]
2018^*^	1.59	0.44	**[0.73, 2.45]**	**1.00**	391.41	54.87	[107.92, 1061.38]
2019^*^	2.03	0.48	**[1.08, 2.98]**	**1.00**	661.94	62.13	[194.63, 1870.55]
2020^*^	3.64	0.61	**[2.44, 4.84]**	**1.00**	3699.67	84.19	[1043.11, 12,530.22]
Male^ǂ^	0.37	0.29	[− 0.40, 1.14]	0.35	44.35	33.59	[− 33.29, 212.34]
Fixes by days	− 0.30	0.21	[− 0.68, 0.07]	0.55	− 26.21	22.95	[− 49.14, 7.04]

b) Average maximum daily displacement (n = 376 estimates from 81 birds)							
Log-transformed model estimates					Back-transformed estimates as % change		
Fixed effects	Estimate	Unc. SE	95% CI	RVI	Estimate	Unc. SE	95% CI
*Intercept*	− 1.17	0.35	[− 1.85, − 0.49]		− 69.02	41.44	[− 84.33, − 38.76]
Juvenile^Ϯ^	− 0.53	0.13	**[**− **0.78, **− **0.29]**	**1.00**	− 41.27	13.34	[− 54.09, − 24.88]
Wild-caught^+^	1.29	0.24	**[0.83, 1.76]**	**1.00**	264.58	26.69	[128.97, 480.50]
Spring^‡^	− 0.29	0.17	[− 0.54, − 0.03]	0.49	− 24.98	18.52	[− 41.81, − 3.28]
Summer^‡^	− 0.18	0.11	[− 0.37, 0.01]	0.49	− 16.31	11.77	[− 30.60, 0.93]
Winter^‡^	− 0.05	0.09	[− 0.31, 0.21]	0.49	− 4.87	9.91	[− 26.32, 22.82]
2018^*^	0.79	0.22	**[0.35, 1.23]**	**1.00**	120.87	25.07	[42.25, 242.94]
2019^*^	0.73	0.24	**[0.25, 1.21]**	**1.00**	107.07	27.51	[28.41, 233.93]
2020^*^	1.03	0.28	**[0.47, 1.59]**	**1.00**	179.25	32.96	[59.49, 388.95]
Male^ǂ^	0.02	0.11	[− 0.41, 0.45]	0.25	2.10	11.64	[− 33.60, 57.00]
Fixes by days	− 0.33	0.12	**[**− **0.54, **− **0.12]**	**0.98**	− 28.03	12.44	[− 41.71, − 11.13]

Reference categories: ϮAge class: ‘adult’, + origin: ‘captive-released’, ‡season: ‘autumn’, *year: ‘2017’, and ǂsex: ‘female’. Predictors with a relative variable importance (RVI) greater that 80% (i.e., 0.8) are highlighted in bold along with the respective confidence intervals (95% CI)

Age class and origin, but not season, were the most important factors explaining average maximum daily displacement (Table 2; Fig. 2d–f). Juveniles had a smaller average maximum daily displacement than adults by − 41.27% [− 54.09, − 24.88]. Wild-caught individuals had a larger average maximum daily displacement than captive-released individuals by 264.58% [128.97, 480.50].

### AFS-use

**Fig. 3:**
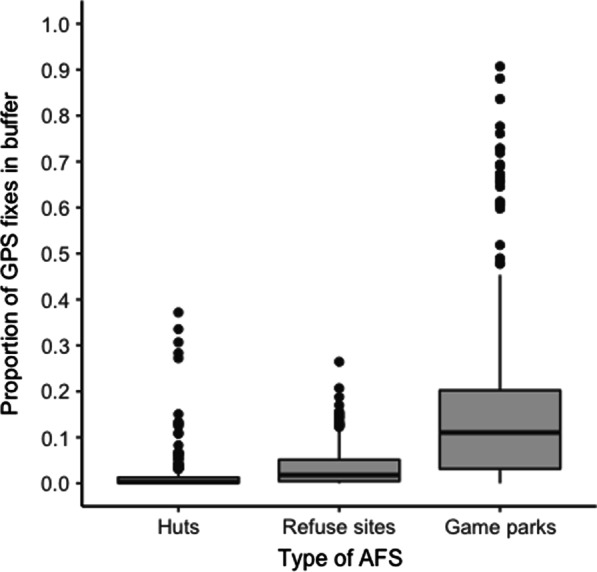
The proportion of GPS-fixes recorded from 79 non-breeding common ravens (*Corvus corax*) within buffers, which were set around AFSs (n = 45) in the Austrian Alps, was greatest for game parks, followed by refuse sites and huts

We analysed resource use at 45 year-round or seasonally available AFSs in Austria, with two refuse sites situated in the Czech Republic just beyond the Northern Austrian border (F10 and F11, Additional file 3; Fig. 1). More than half of the sites (n = 29) were visited by 5 or fewer tracked individuals, 10 sites were visited by 6 to 20 individuals, and only 6 sites were visited by more than 20 individuals. The most popular site, Cumberland Wildpark (F01, game park) was the location where all wild-caught ravens were trapped. Given that captive-bred individuals were released at the KLF aviary approximately 700 m away, all tracked individuals in this study had knowledge of this game park and most of them also returned to it (see [71] for another example of familiar foraging site preference). In comparison, the second and third most popular sites were a compost site (F02) and an alpine hut (F36), both attracting 45 individuals which are both close in distance to the game park (Additional file 3). These trends reflected in the proportion of points recorded inside AFS buffers by resource type, with values being the highest for game parks, followed by refuse sites and huts (Fig. 3).

On average, per season and year, individuals visited 2.6 ± 0.1 AFSs (0–11 AFSs, n = 376), and had 19.03 ± 0.84% (0.53–90.73%, n = 369) of their total fixes located within a single buffer, indicating high site fidelity.

**Fig. 4:**
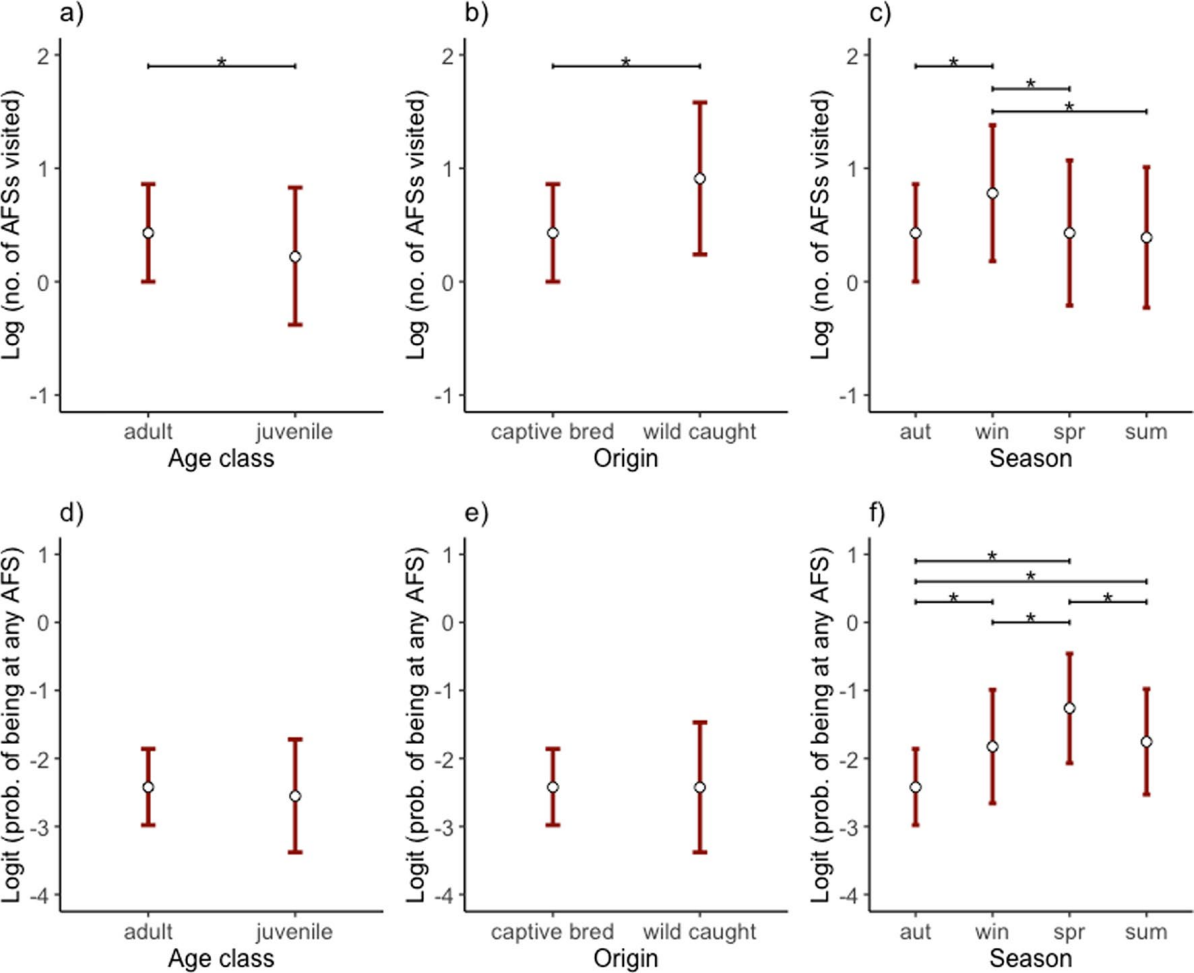
The number of AFSs visited (presented on a logarithmic scale) by 81 GPS-tagged non-breeding common ravens (*Corvus corax*) in the Austrian Alps differed between **a** adults and juveniles, **b** captive-released and wild-caught individuals and **c** seasonally. The probability of being at any AFS (presented on a logit scale), determined by the proportion of GPS-fixes recorded within buffers that were set around AFSs (n = 79 individuals), did not differ between **d** adults and juveniles, **e** captive-released and wild-caught individuals but only **f** seasonally. White circles with red error bars depict model-averaged estimates from a generalised linear mixed model (GLMM), with 95% confidence intervals (Table 3)

There was a strong effect of age, origin and season on the number of AFSs visited by individuals per season and year (Table 3; Fig. 4a–c). Juveniles visited fewer sites than adults by − 18.55% [− 31.65, − 2.94], and wild-caught individuals visited more sites than juveniles by 61.67% [27.15, 105.56]. Relative to autumn, the number of AFSs visited was higher in winter by 42.50% [20.25, 68.87], and lower in spring and summer by − 0.12% [− 19.16, 23.41] and − 3.85% [− 20.32, 16.02] respectively, with differences noted between winter and the other seasons (Additional file 7).

**Table 3:** Model averaging outputs for the top models based on ∆AICc <  = 6 for (a) the number of anthropogenic food sources (AFSs) visited (Poisson error distribution) for 81 individual ravens, and (b) the probability of an individual being at any AFS (binomial error distribution) for 79 individual ravens GPS-tagged for 2.75 years in the Austrian Alps

a) Number of AFSs visited (n = 376 estimates from 81 birds)							
Log-transformed model estimates					Back-transformed estimates as % change		
Fixed effects	Estimate	Unc. SE	95% CI	RVI	Estimate	Unc. SE	95% CI
*Intercept*	0.43	0.22	[0.00, 0.86]		53.53	− 78.17	[0.00, 135.71]
Juvenile^Ϯ^	− 0.21	0.11	**[**− **0.38, **− **0.03]**	**0.86**	− 18.55	− 89.10	[− 31.65, − 2.94]
Wild-caught^+^	0.48	0.12	**[0.24, 0.72]**	**1.00**	61.67	− 87.78	[27.15, 105.56]
Spring^‡^	0.00	0.11	**[**− **0.21, 0.21]**	**1.00**	− 0.12	− 89.24	[− 19.16, 23.41]
Summer^‡^	− 0.04	0.10	**[**− **0.23, 0.15]**	**1.00**	− 3.85	− 90.44	[− 20.32, 16.02]
Winter^‡^	0.35	0.09	**[0.18, 0.52]**	**1.00**	42.50	− 91.36	[20.25, 68.87]
2018^*^	0.34	0.17	[− 0.22, 0.91]	0.15	41.16	− 83.35	[− 19.47, 147.43]
2019^*^	0.40	0.18	[− 0.18, 0.98]	0.15	49.50	− 81.55	[− 16.16, 166.59]
2020^*^	0.31	0.17	[− 0.34, 0.96]	0.15	36.10	− 83.03	[− 28.80, 160.17]
Male^ǂ^	0.07	0.06	[− 0.13, 0.28]	0.29	7.66	− 93.55	[− 11.96, 31.66]
Fixes by days	− 0.04	0.05	[− 0.22, 0.14]	0.26	− 4.13	− 95.03	[− 19.84, 14.65]

b) Probability of being at any AFS (n = 369 estimates from 79 birds)							
Logit-transformed model estimates					Back-transformed estimates as probability		
Fixed effects	Estimate	Unc. SE	95% CI	RVI	Estimate	Unc. SE	95% CI
*Intercept*	− 2.42	0.29	[− 2.98, − 1.86]		0.08	0.57	[0.05, 0.13]
Juvenile^Ϯ^	− 0.13	0.10	[− 0.40, 0.14]	0.36	0.07	0.53	[0.03, 0.15]
Wild-caught^+^	0.00	0.10	[0.39, 0.39]	0.26	0.08	0.53	[0.03, 0.19]
Spring^‡^	1.16	0.12	**[0.92, 1.40]**	**1.00**	0.22	0.53	[0.11, 0.39]
Summer^‡^	0.67	0.11	**[0.45, 0.88]**	**1.00**	0.15	0.53	[0.07, 0.27]
Winter^‡^	0.60	0.14	**[0.32, 0.87]**	**1.00**	0.14	0.53	[0.07, 0.27]
2018^*^	0.58	0.26	**[0.07, 1.09]**	**1.00**	0.14	0.56	[0.05, 0.32]
2019^*^	− 0.04	0.28	**[**− **0.58, 0.50]**	**1.00**	0.08	0.57	[0.03, 0.20]
2020^*^	− 0.09	0.34	**[**− **0.75, 0.57]**	**1.00**	0.07	0.58	[0.02, 0.22]
Male^ǂ^	− 0.04	0.09	[− 0.39, 0.31]	0.26	0.08	0.52	[0.03, 0.18]

Reference categories: ϮAge class: ‘adult’, + origin: ‘captive-released’, ‡season: ‘autumn’, *year: ‘2017’, and ǂsex: ‘female’. Predictors with a relative variable importance (RVI) greater that 80% (i.e., 0.8) are highlighted in bold along with the respective confidence intervals (95% CI)

Only season had high relative variable importance in explaining the probability of being at any AFS (Table 3; Fig. 4d–f). Compared to autumn individuals had higher probabilities of being at any AFS in spring (0.22 [0.11, 0.39]), summer (0.15 [0.07, 0.27]) and winter (0.14 [0.07, 0.27]. The probability of being at any AFS in spring was notably higher than all the other seasons, and in in summer and winter relative to autumn (Additional file 7).

## Discussion

In this study, we found that non-breeding ravens exhibit pronounced differences in their movement patterns based on age, origin, and the seasonality of the environment. An individual’s age (i.e., adult versus juvenile) and early life experience (i.e., wild-caught versus captive-released) strongly predicted its movement patterns and the number of AFSs visited. Adults and wild-caught birds reported a higher average occurrence distribution, maximum daily displacement, and number of AFSs visited than juveniles and captive-released birds. Seasonal changes in environmental conditions explained patterns in occurrence distribution, but not the maximum daily displacement, and in both AFS-use metrics. We illustrate how movement data can be used to characterize foraging behaviour, and how non-breeding ravens exploiting AFSs exhibit differences in foraging strategies.

### Differences in age and origin cohorts

Our results show that individuals exhibit different movement and foraging patterns linked to their experience and potentially dominance status. We inferred both through the fitted predictors age and origin, which unravelled that juveniles and captive-released individuals exhibit less vagrancy (i.e., lower occurrence distribution and shorter average maximum daily displacement) and visit fewer AFSs than adults and wild-caught birds. These cohort differences, however, did not affect the probability of individuals being at any food source, suggesting that individuals are equally likely to exploit AFSs. This finding substantiates existing evidence on the importance of AFSs for non-breeders in this population [23]. We found no difference in movement or foraging behaviour between males and females, supporting previous studies on ravens’ space use [23, 42, 47] and foraging proficiency [72].

Juveniles and captive-released birds (who were released as juveniles) might lack familiarity with feeding conditions [72], knowledge of surrounding AFSs, and flying efficiency [73, 74], therefore opting for a “local” or less exploratory foraging strategy [71]. In doing so, these individuals could benefit from developing familiarity with their social and spatial environment [14, 41, 75, 76], increasing their experience and establishing social relationships before investing more in exploratory behavior [15, 33, 77]. With increased learning and memory, birds may also lower risks and costs associated with travelling across unknown areas and exploiting the resources there [11, 15, 31].

Juveniles and captive-released birds typically rank low in social settings [40, 48], and the limited exploratory behavior exhibited may reflect them being spatially limited in their access to food resources. At small and clumped food sources, high-ranking individuals are able to monopolize and defend resources from conspecifics [46]. Conversely, the quantity and distribution of food at large, and often permanent, food sources enable less-dominant individuals to neutralize defenses and access the resource despite high-ranking individuals [14, 28]. These communally exploited AFSs may therefore attract more low-ranking individuals than non-communal sites [46]. Furthermore, at communal AFSs, subordinate birds benefit from having power in numbers to overcome defenses at food resources [78], however this is not necessarily the case in other soaring scavengers [79, 80]. In contrast, adults and wild-caught birds that are more experienced with the landscape could mitigate high levels of competition by searching for and exploiting non-communal AFSs [33, 49, 72]. With age, exploration may also increase as individuals search for partners and territories [81, 82]. For management strategies aimed at translocating individuals away from problem areas, targeting less vagrant juveniles, who are also easier to capture [39], may prove to be more effective than wide-ranging adults.

### Seasonal differences

Seasonal changes in environmental conditions and food availability might reflect harshness or ecological favourability for species and result in shifting foraging tactics of individuals [30]. In temperate climates, natural food source availability decreases starting autumn through winter, and increases once again in spring. Avian scavengers, like ravens, can experience higher food-searching costs in low temperatures, high snow cover (which hides natural food sources), short day lengths, and limited opportunities for good flight conditions (e.g., fewer thermal uplifts in winter reduces flight distance and duration) [23, 31, 39, 49, 79]. When conditions get warmer, anthropogenic food availability in temperate regions can change as large amounts of garbage melt out of the snow [32], and as organic waste is raked out of barns to compost in the warming weather. Natural food sources such as insects, grain, and carcasses that appear from below avalanches also become more available. When coping with such changes, predictable AFSs may represent comparatively abundant and attractive resources to forage at [79, 81]. Our results showed that while birds maintain a similar average maximum daily displacement across all seasons, they varied in terms of how widely they dispersed across the landscape and in their AFS-use.

An increase in energy demands in colder months may prompt birds to search larger areas for AFSs or natural food. Birds had the largest occurrence distributions in autumn followed by winter, and visited the highest number of AFSs in winter. While flying can be energetically expensive, the benefits reaped from finding and exploiting food sources are likely to be greater than the costs [19], however this is may not always be the case [80]. In these seasons, AFSs may also become more desirable as shorter daylight reduces the time available for foraging. Additionally, flying in search for food could mitigate the competition that arises from many individuals relying heavily on a limited amount of resource under stressful conditions. Time spent moving between AFSs and the use of natural food sources reduces the time spent directly at AFSs, which would explain why contrary to expectation, the probability of individuals being present at any AFS was found to be low in autumn and winter.

The combination of wide-ranging movements, yet a lower number of AFSs visited in autumn may be attributed to the availability of ephemeral carcasses distributed across the landscape from the hunting seasons (in Austria: 1st August to 31st December for stag and 1st May to 31st October for roe deer and chamois, with small regional differences regarding dates). Although carcasses are also present in winter, seasonal AFSs such as skiing huts become available with the winter tourism season in the Alps. The large occurrence distribution and high number of AFSs visited in winter may therefore reflect both an increased availability of and dependency on AFSs. The heightened winter-time dependency on AFSs is further supported by studies in Greenland [49], the Austrian and Bavarian Alps [23, 83], Alaska [32], North Finland [84] and North Wales [76], that have all noted increases in the number of ravens present at AFSs in winter. Similar observations exist for other species that exploit anthropogenic resources to a larger extent during the harsher winter months such as Kea *Nestor notabilis* [85], Bald Eagles *Haliaeetus leucocephalus* [86, 87] and White storks *Ciconia ciconia* [88]. Given the seasonality in AFS dependency exhibited by ravens, targeted winter-time food closures could provide an effective non-lethal management strategy to control population growth. In systems where population reduction is desirable, season-based management [87] can decrease the fitness of juveniles who depend greatly on abundant food to survive through their first winter [78].

The number of AFSs visited in summer and spring were only slightly lower than in autumn despite the occurrence distributions being notably smaller. Like in autumn, the presence of more natural food sources (i.e., carcasses, insects, grain) and absence of wintertime AFSs might explain why the number of AFSs visited is comparable. Small occurrence distributions in summer and spring may result from differences in foraging and energetic requirements to winter and autumn, and may also be suggestive of breeders becoming highly territorial. Although breeders maintain territories throughout the year, they move extraterritorially especially in autumn and winter to access food resources [25, 42]. In the breeding season, which commences in late winter, through spring, and until summer, breeding pairs become highly territorial. The breeders’ rank is highest in social dominance and in a landscape saturated with breeding territories, non-breeders might be limited in their movements [25, 42]. Furthermore, the limited time spent by breeders at AFSs during the breeding season decreases competition [71], possibly explaining why the probability of being present at any AFS was the highest in spring.

## Conclusions

In this study, we used broad-scale movement patterns, inferred from high resolution spatial and temporal data, of non-breeding ravens and showed that movement and resource-use differ according to individual traits and the seasonality of the environment. The age and origin cohort differences highlight how predictable AFSs can shape foraging strategies among non-breeding ravens. Seasonal differences reveal how non-breeding ravens exploit AFSs under complex environmental conditions. Ravens can be heavily nomadic during their non-breeding life-stage [53], yet we observed them to move around and frequently revisit AFSs in our study region. The extent of AFS-exploitation among non-breeding ravens in our study emphasize the potential of AFSs in shaping raven movement and resource-use.

## Supplementary Information

**Additional file 1**. General information on the 81 individuals (ID, Name) that were tracked for 2.75 years in the Austrian Alps, including age class, season, sex (m = male, f = female), origin and death comment. The number of tracking days, fixes, the occurrence distribution (calculated as the 95% contour area of the utilization distribution or 95% UD), the average maximum daily displacements, the number of AFSs visited and the proportion of GPS fixes inside a buffer are presented for each individual-season-year combination (n = 376).

**Additional file 2**. Examples of Google Earth satellite imagery of the identified anthropogenic food sources in the Austrian Alps.

**Additional file 3**. Information on the 45 anthropogenic food sources. Broader resource type groupings (refuse, hut, game park) were based on similarities in sites that were verified (Y) and described from the field surveys. Buffers were estimated based on revisitation analysis, at the distance where revisitations no longer increased with increasing radius size or at the maximum of 150 metres (Additional file 4). The number of different individuals (No. Ind) that were recorded at a site across the tracking period is also reported.

**Additional file 4**. Revisitation plots for 45 anthropogenic food source sites exploited by common ravens in the Austrian Alps.

**Additional file 5**. The top-ranking models for **(a) **the occurrence distribution, **(b) **average maximum daily displacement, **(c)** the number of anthropogenic food sources (AFSs) visited and **(d)** the probability of being at any AFS of raven, *Corvus corax*, individuals tracked in the Austrian Alps from a cut-off at ΔAICc ≤ 6 (Akaike’s Information Criterion corrected for small sample sizes). The global model for all response variables included sex, origin (i.e., wild-caught, captive-released), age class (i.e., juvenile, adult), season (i.e., autumn, winter, spring, summer) and year as fixed factors. Models (a), (b) and (c) included the ratio of the number of GPS fixes to tracking days for each individual-season combination as a fixed factor. Individual identity was included as the random factor in all models. We included an additional observation-level random effect (ORLE) in model (d) to account for overdispersion. The number of model parameters (Df), log-likelihood (logLik), AICc, ∆AICc, and model weights (ώ_i_) are presented.

**Additional file 6**. Spatial distribution of the 81 GPS tagged ravens appearing in the dataset in the Austrian Alps. The coloured polygons represent the different individuals’ 95% occurrence distributions in relation to the location of 45 anthropogenic food sources that were identified in this area, grouped by resource type. Major cities are indicated as white and black circles. The box in the upper left corner shows the field site in Austria and the countries neighbouring Austria. Thick black lines show the country borders.

**Additional file 7**. Model averaging outputs for the top models based on ∆AICc <= 6 for **(a) **the occurrence distribution (log-normal distribution), for 81 individual ravens, and **(b)** the probability of an individual being at any AFS (binomial error distribution) for 79 individual ravens GPS-tagged for 2.75 years in the Austrian Alps with different intercept levels for the categorical predictor ‘season’.

## Data Availability

The datasets supporting the conclusions in this article are available in the Movebank Data Repository (10.5441/001/1.22nd28v7), and are included within the article and its additional files.

## Abbreviations

AFSs: Anthropogenic food sources
a. s. l.: Above sea level
KLF: Konrad Lorenz Research Centre
SD: Standard deviation
UD: Utilisation distribution
dBBM: Dynamic Brownian bridge movement
GLMM: Generalised linear mixed model
VIF: Variance inflation factor
AICc: Akaike’s information criterion corrected for small sample sizes
Unc SE: Unconditional standard error
CI: Confidence interval
