# Supplementary material for: The spatial and temporal exploitation of anthropogenic food sources by common ravens (Corvus corax) in the Alps
Source: Mov Ecol. 2022 Aug 25;10:35. doi: 10.1186/s40462-022-00335-4 (PMC9414151; doi:10.1186/s40462-022-00335-4)
Supplement: Supplementary file 4 — Additional file 4. Revisitation plots for 45 anthropogenic food source sites exploited by common ravens in the Austrian Alps. [file 40462_2022_335_MOESM4_ESM.docx]

**Additional file 4** Revisitation plots for 45 anthropogenic food source sites exploited by common ravens in the Austrian Alps.


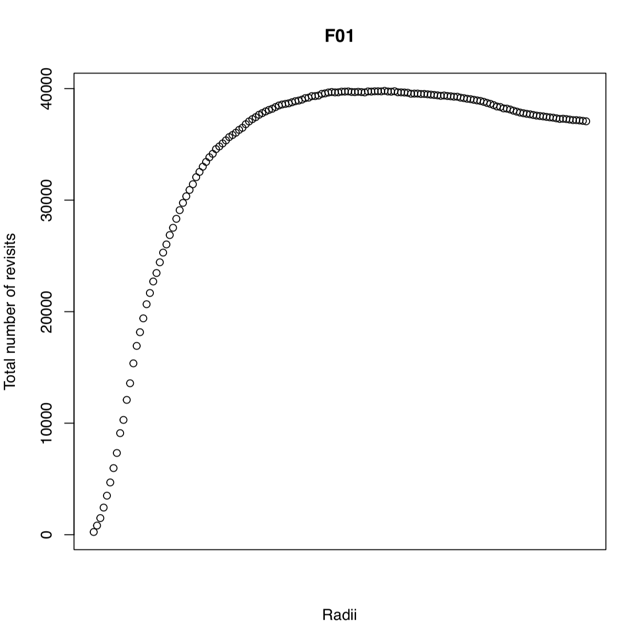

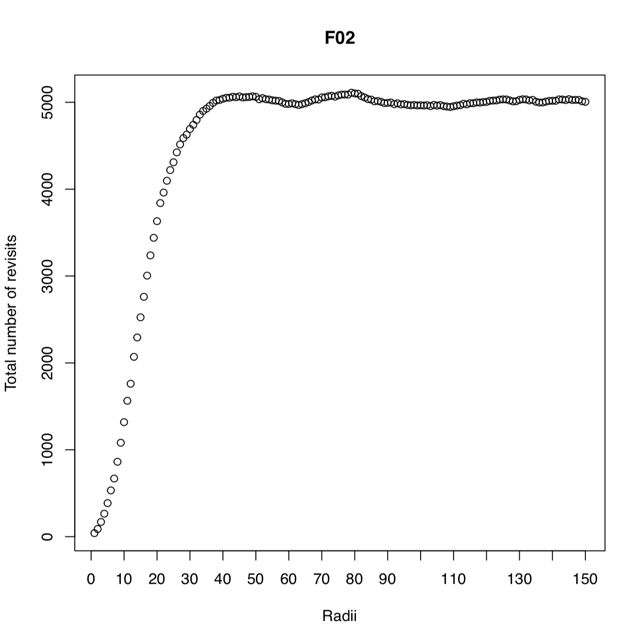

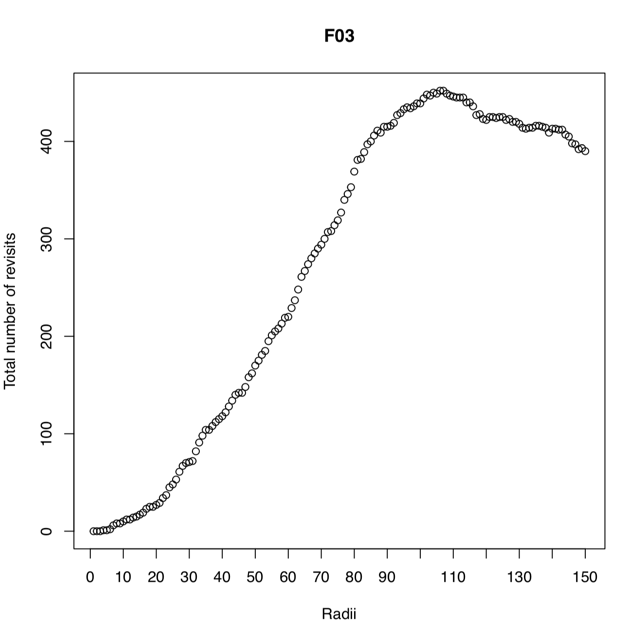
 **
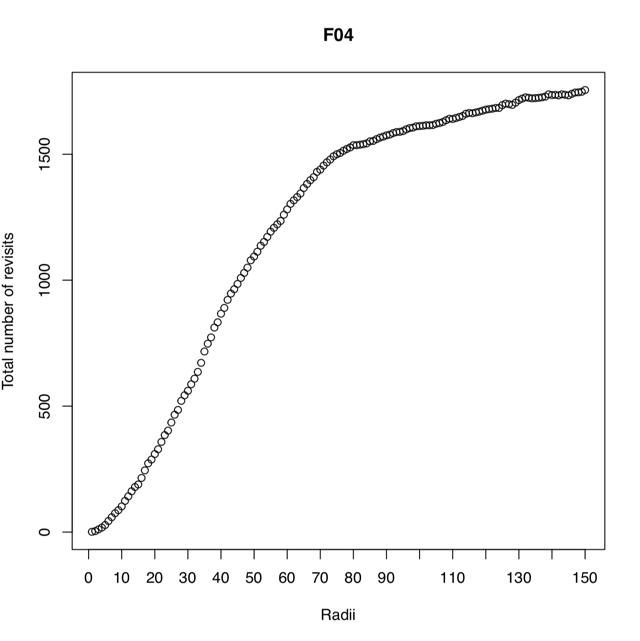

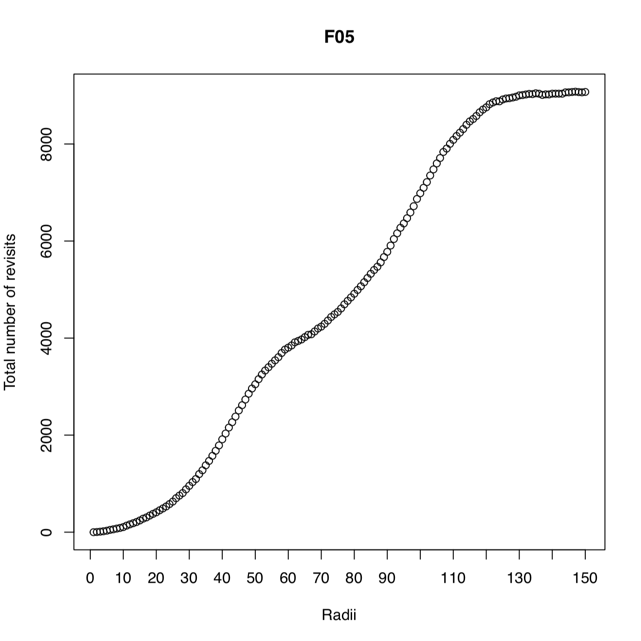

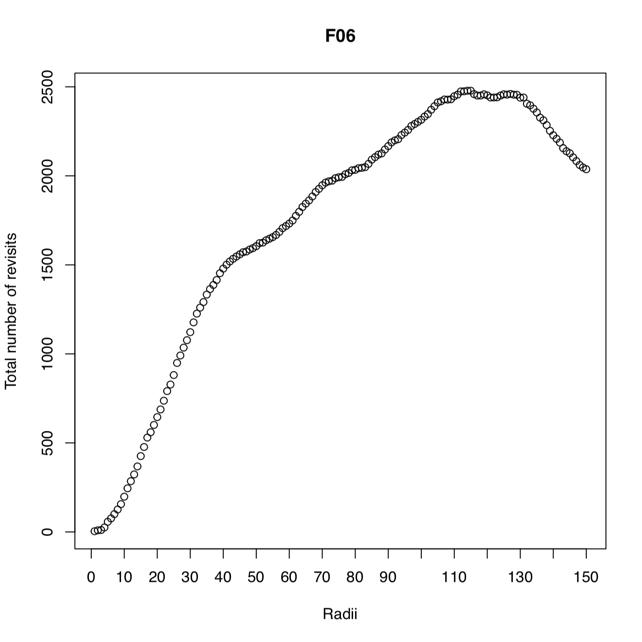

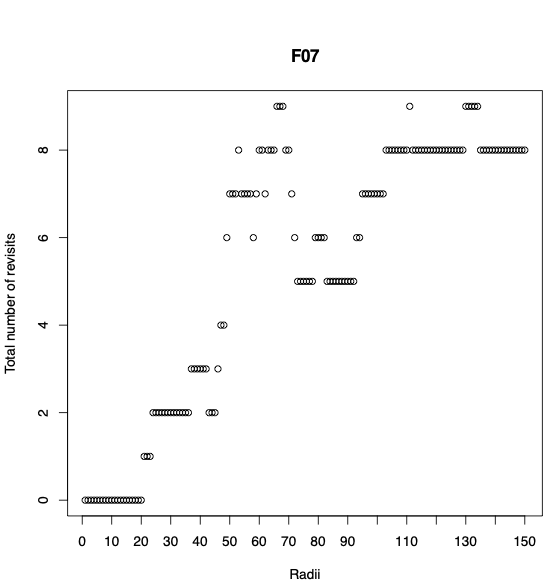

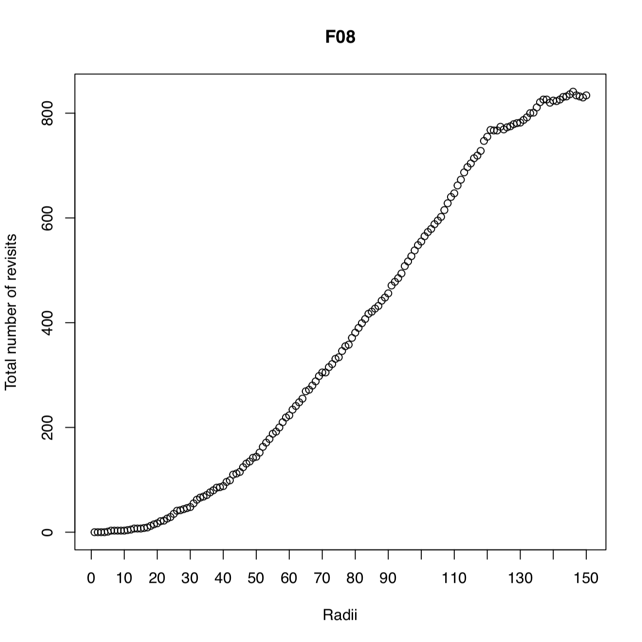

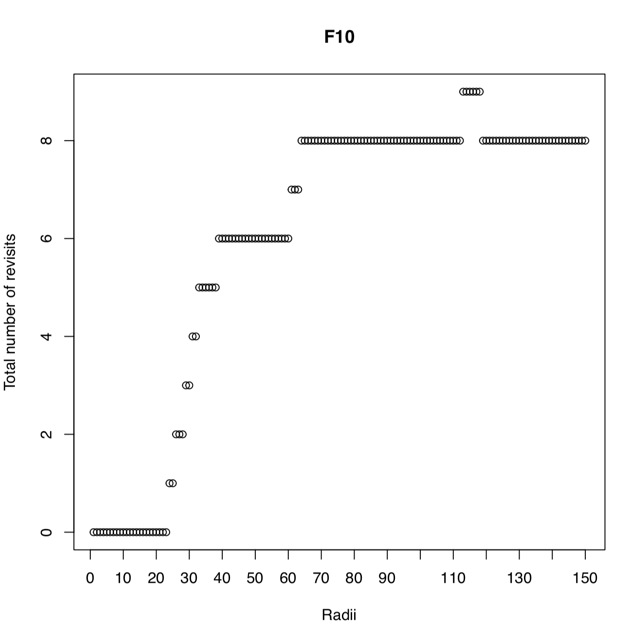

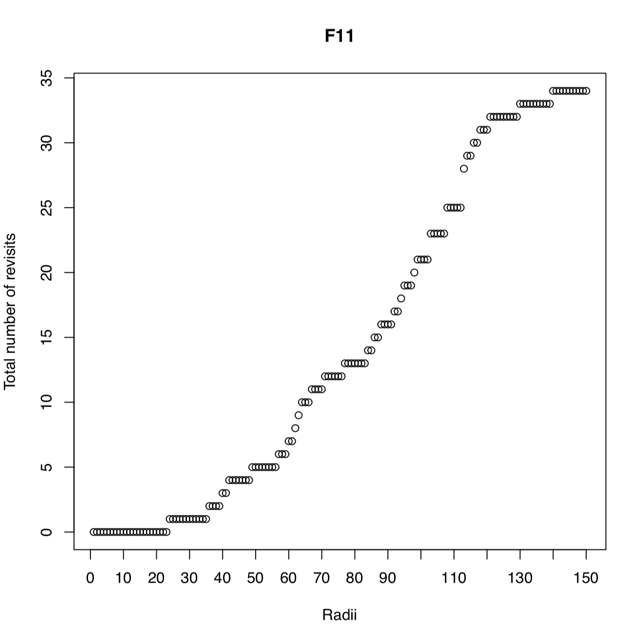

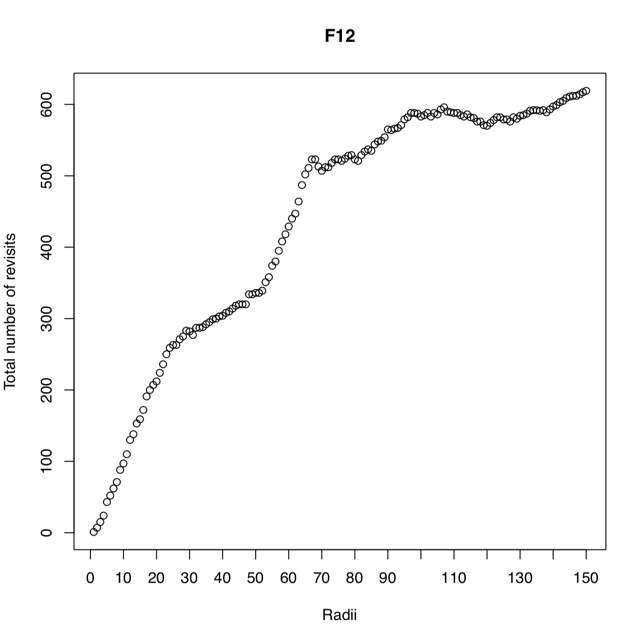

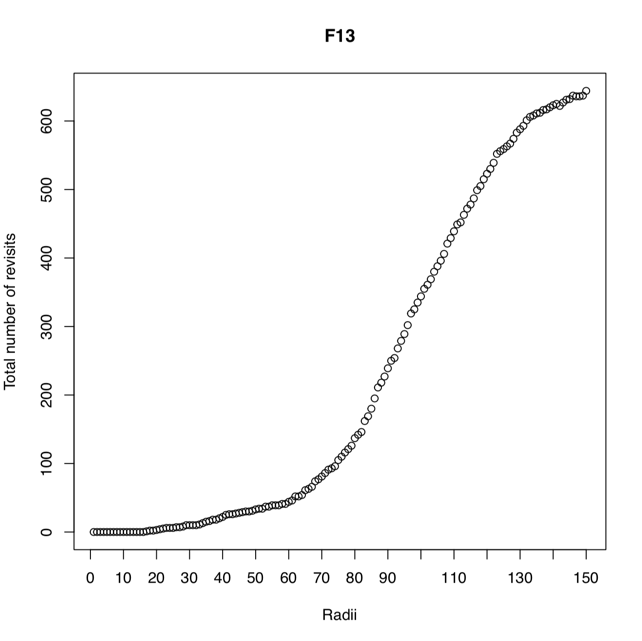

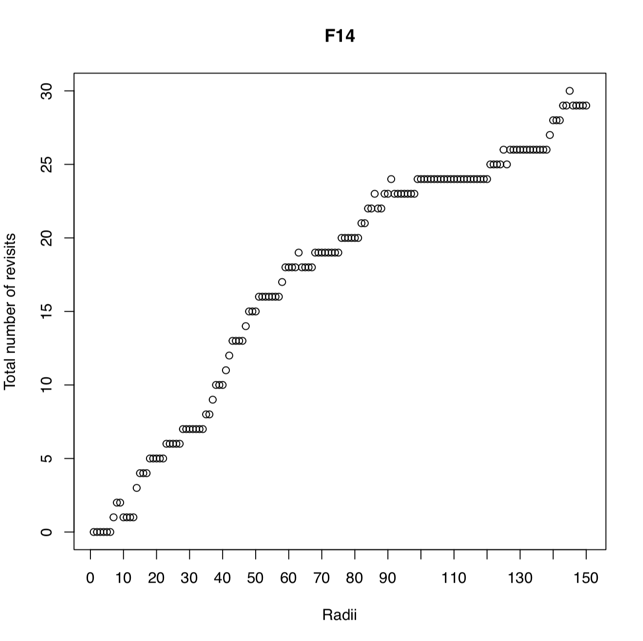

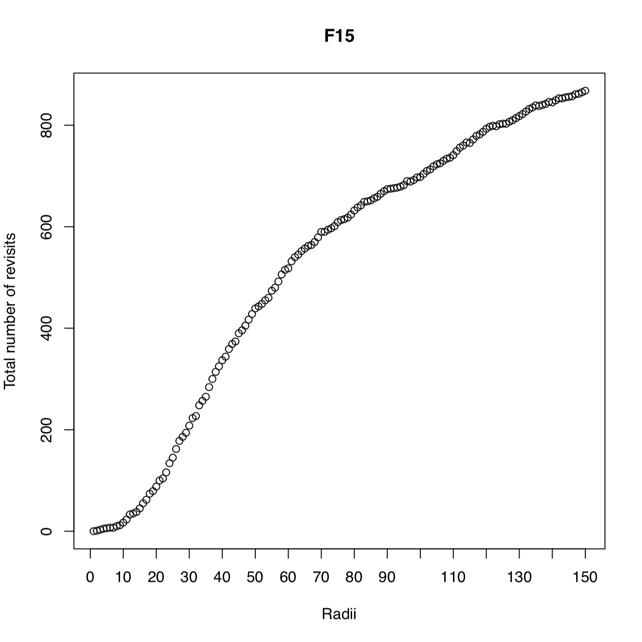

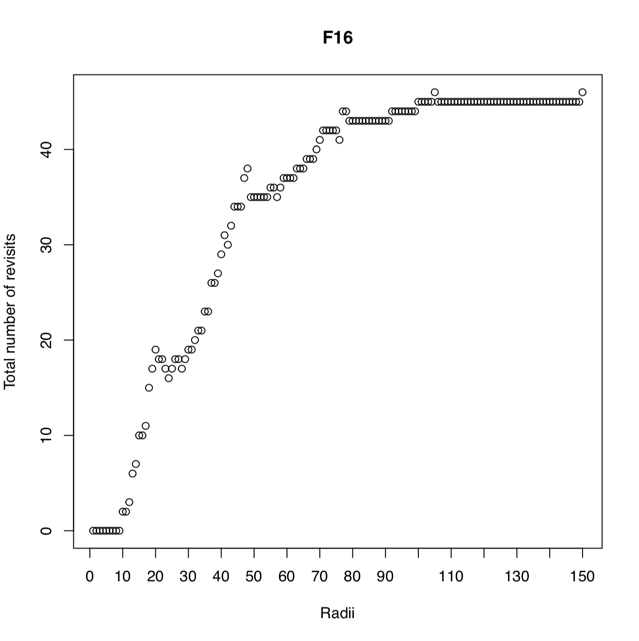

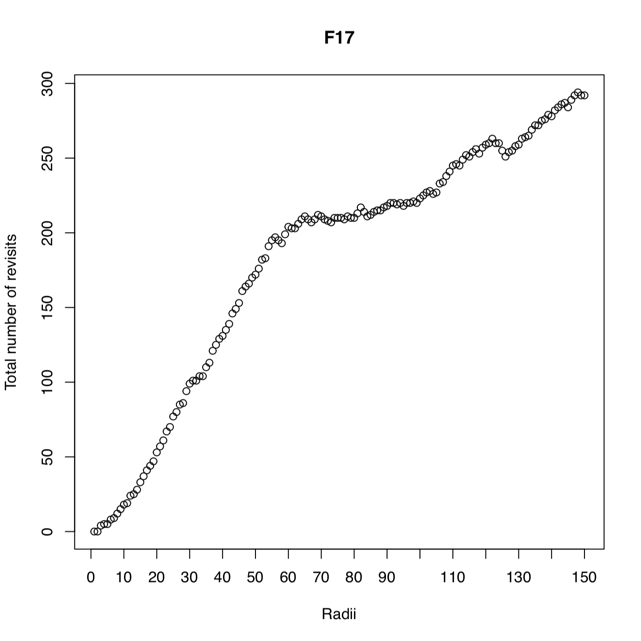

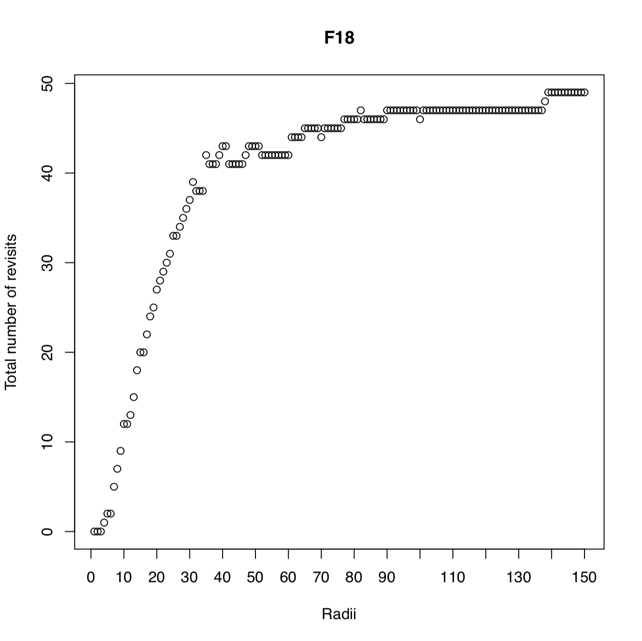

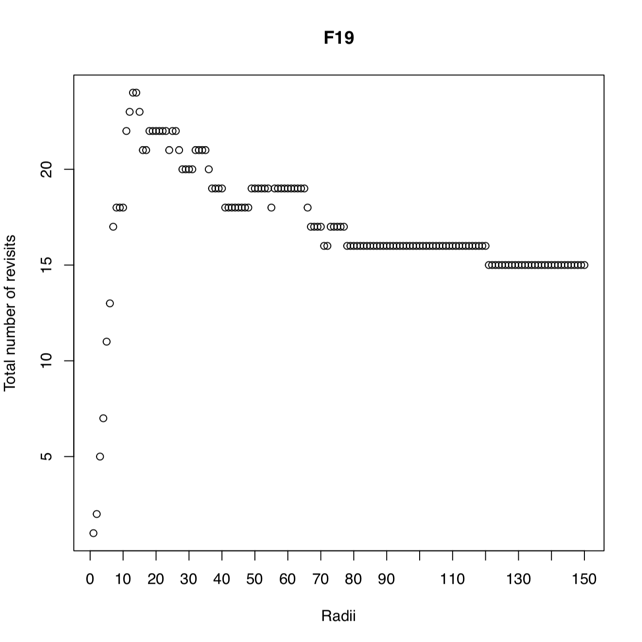

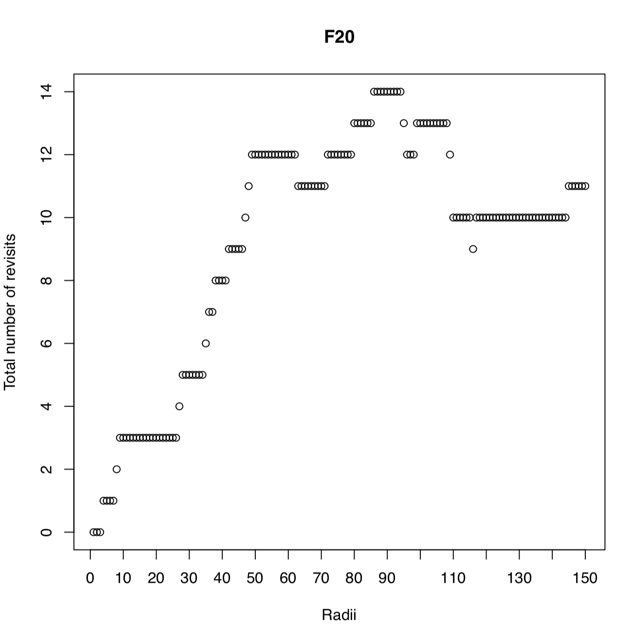

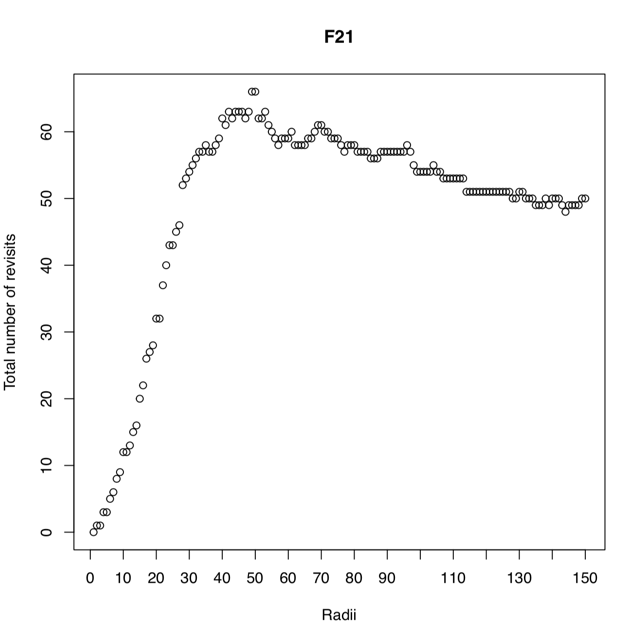

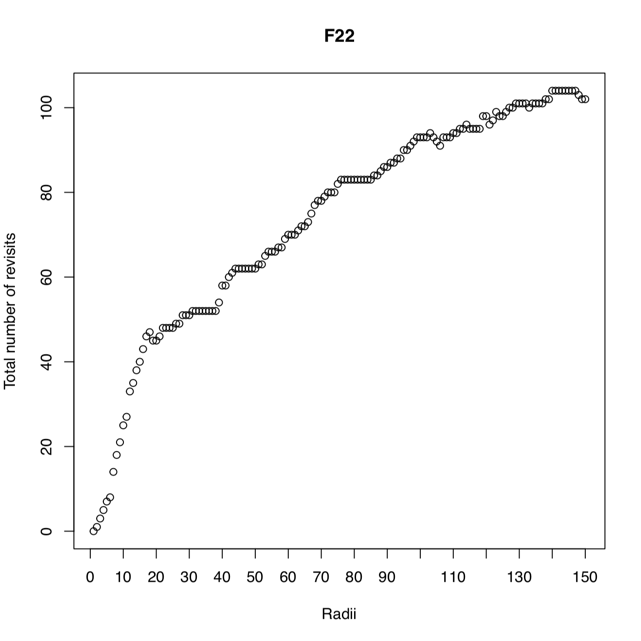

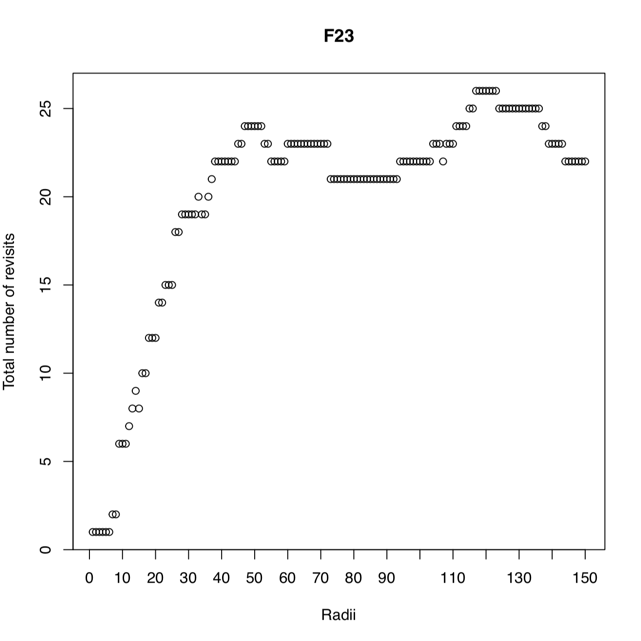

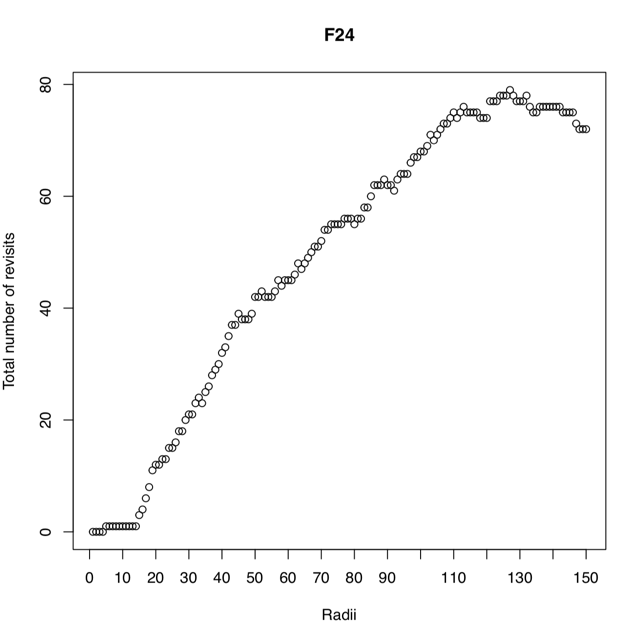

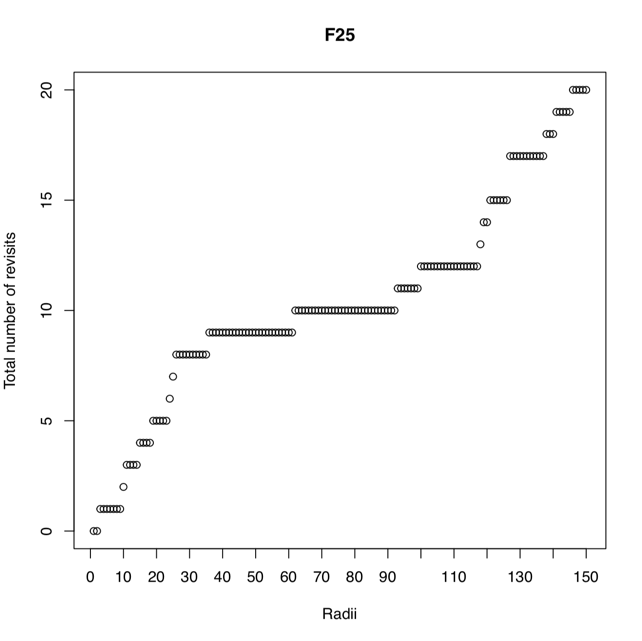

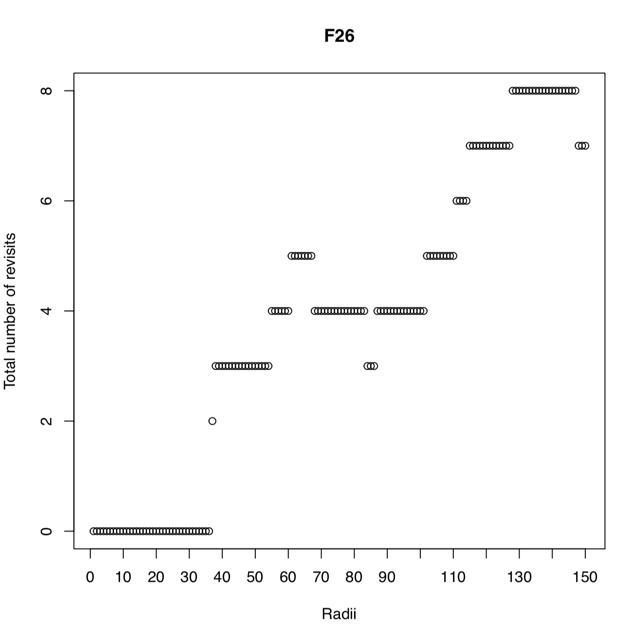

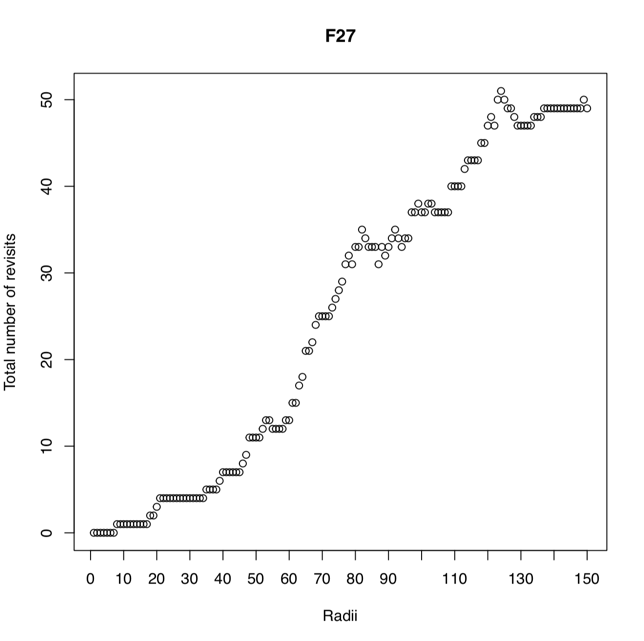

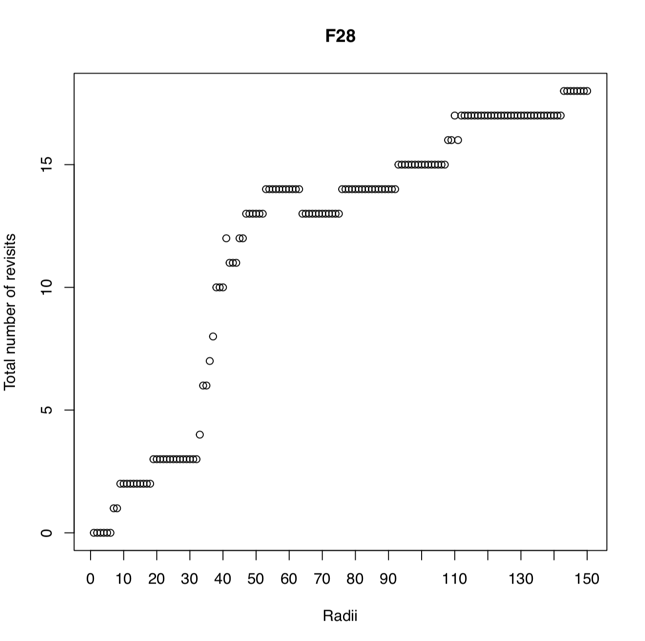

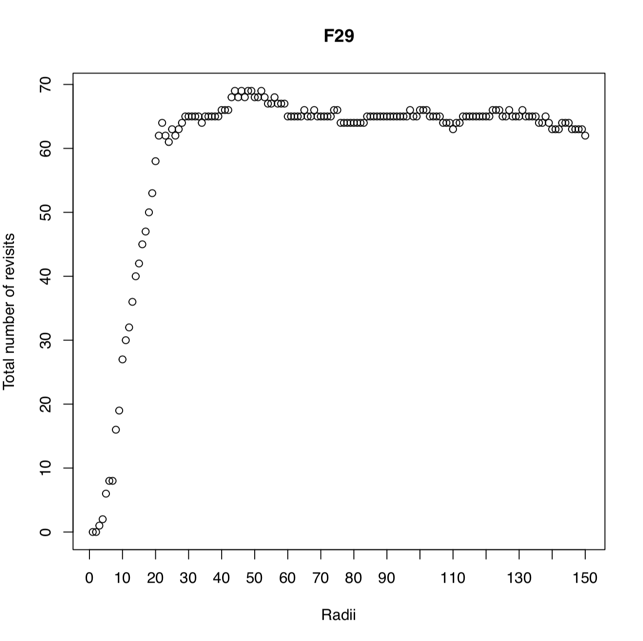

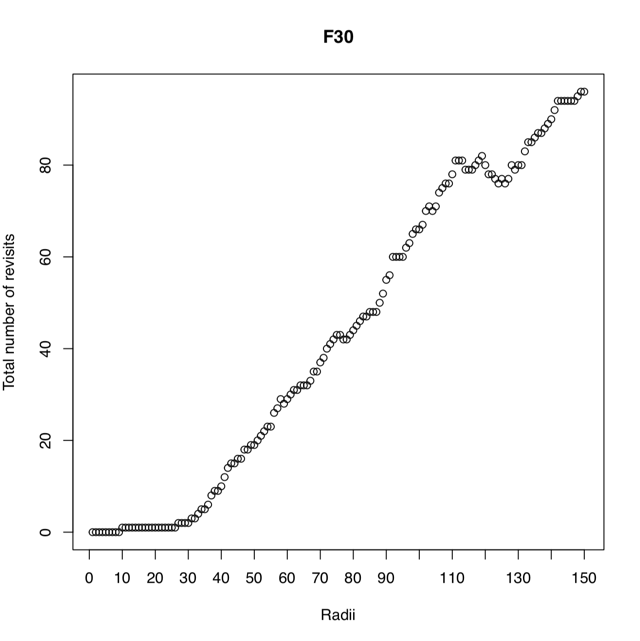

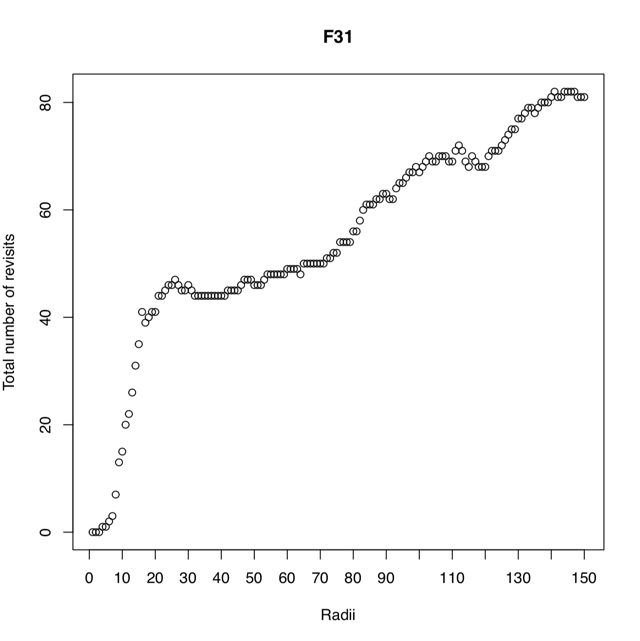

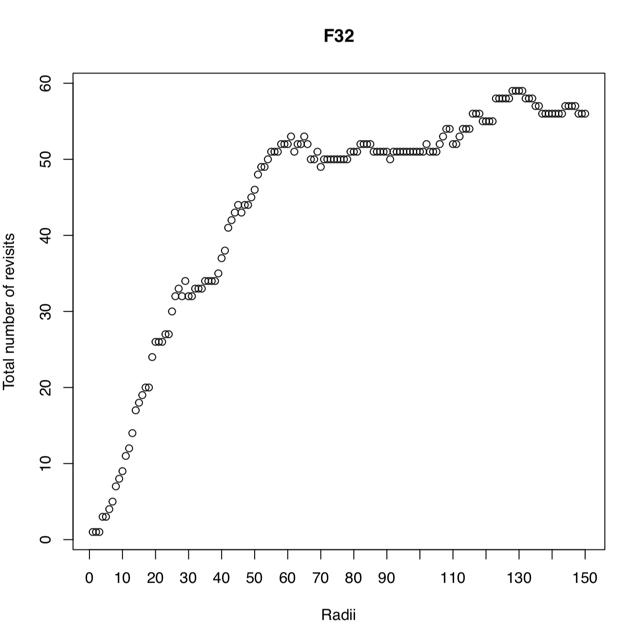

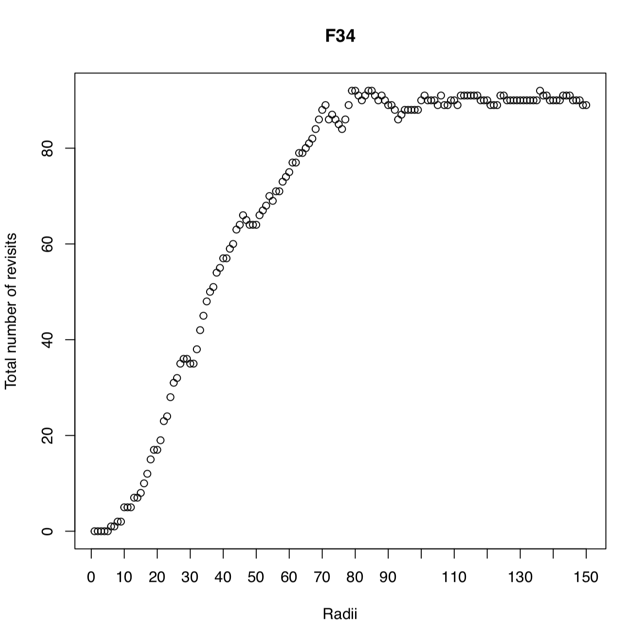

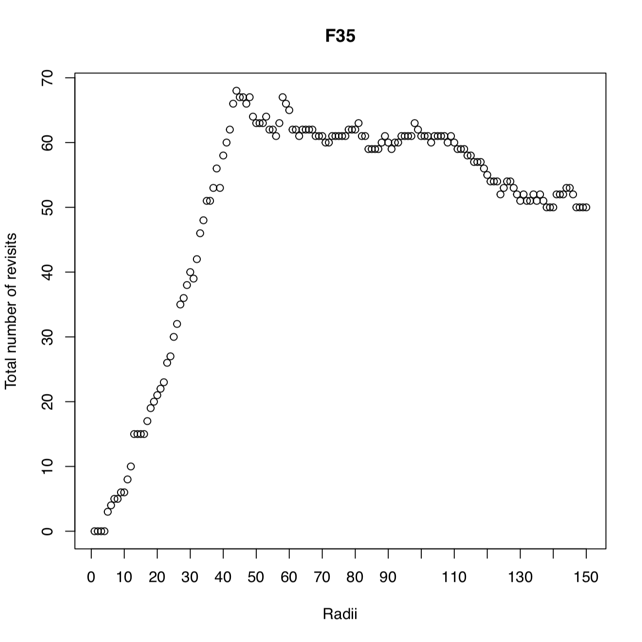

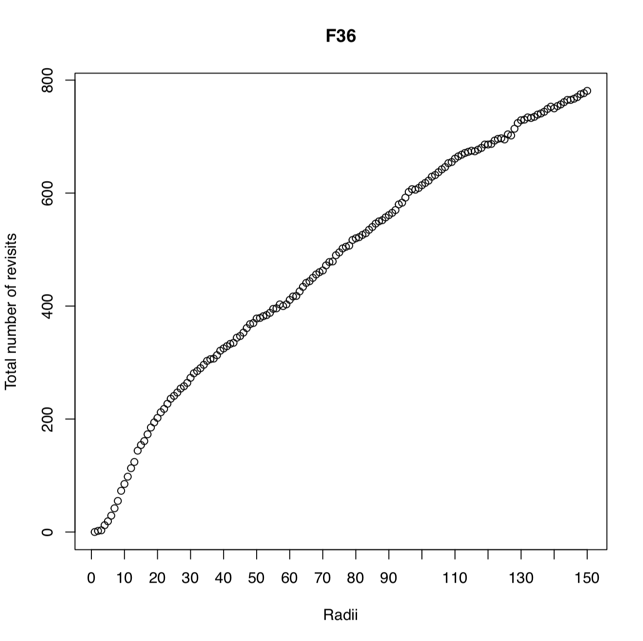

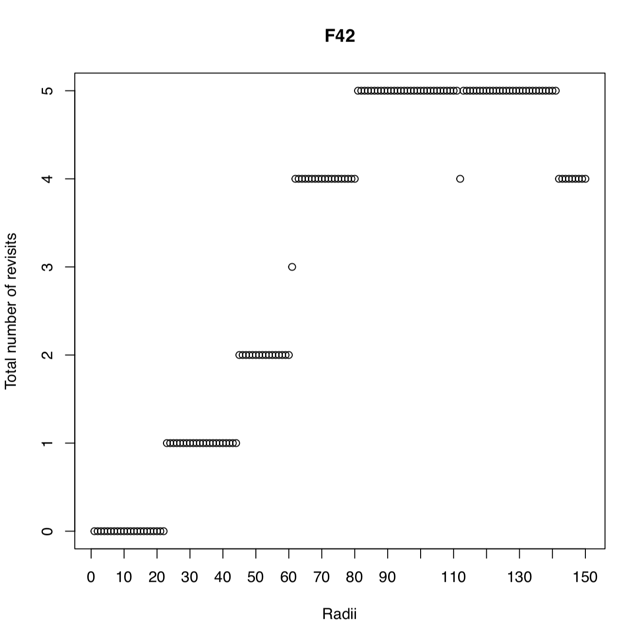

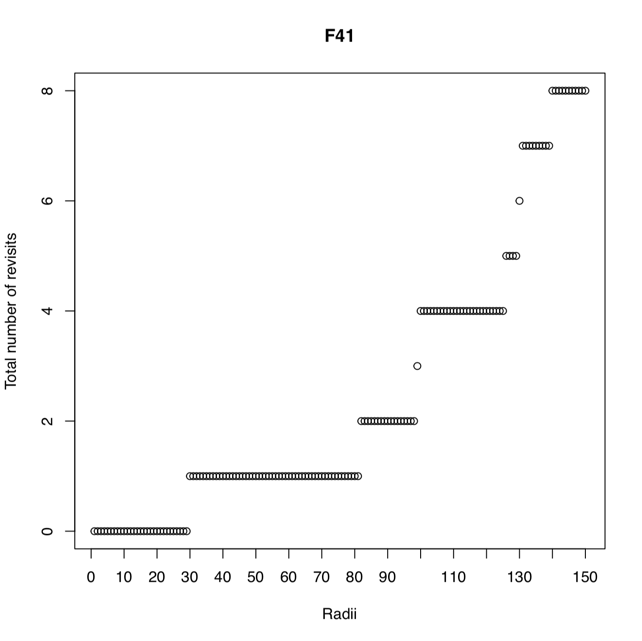

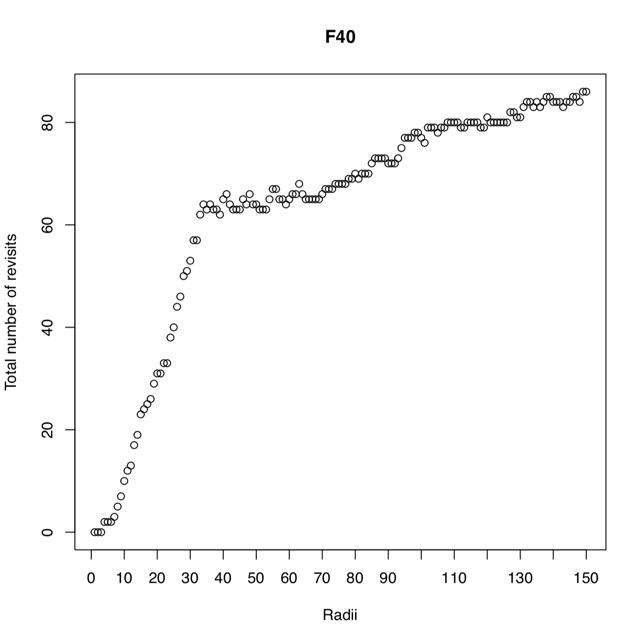

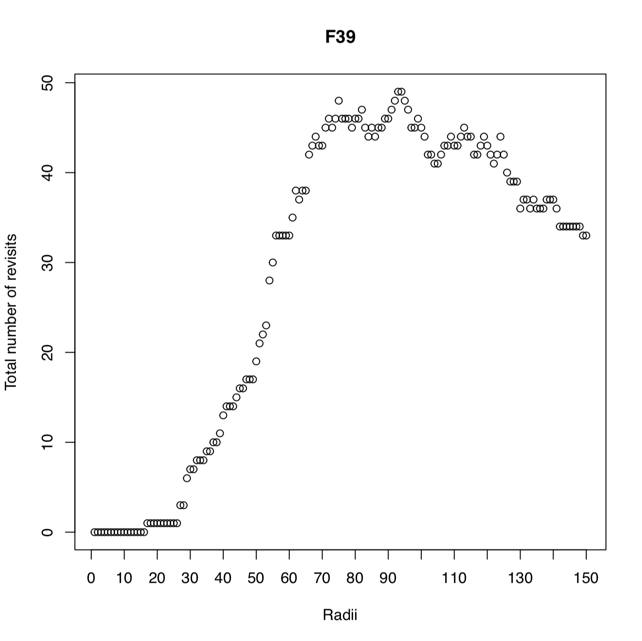

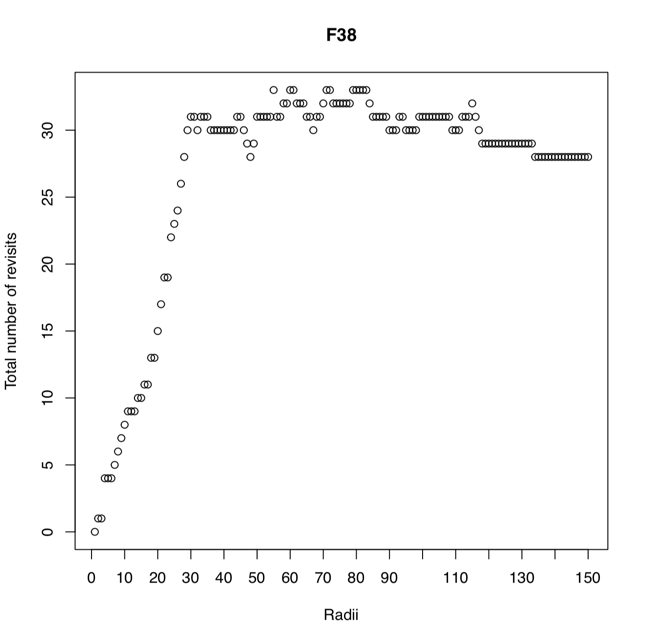

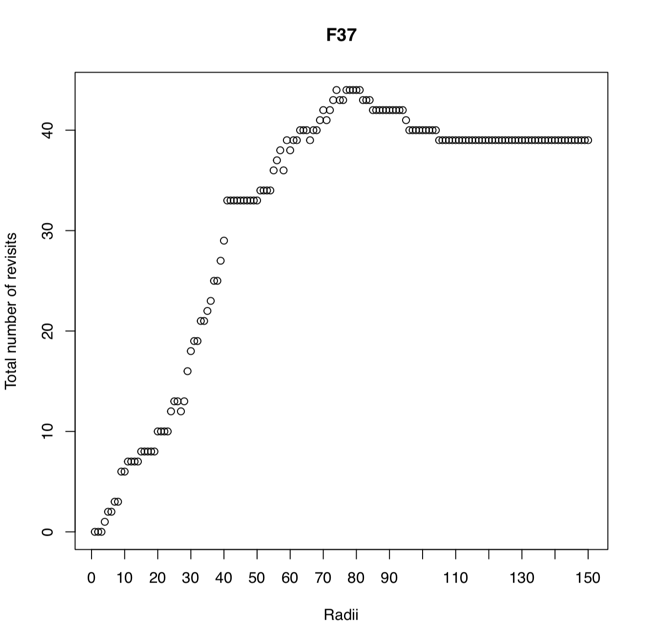

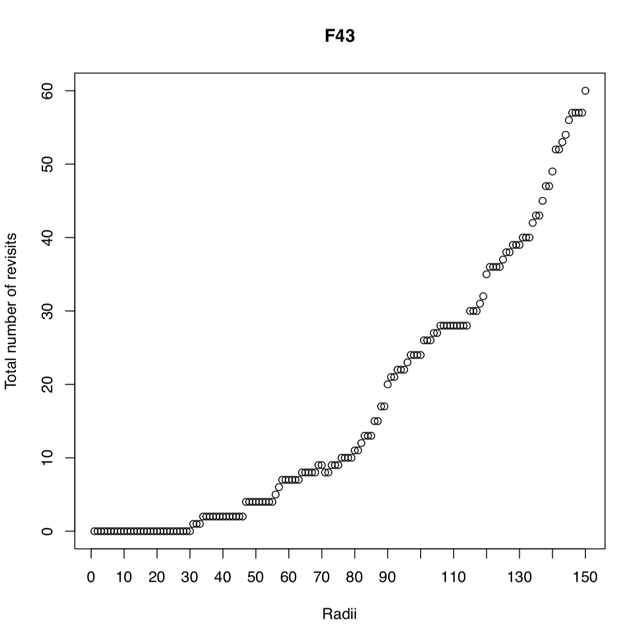

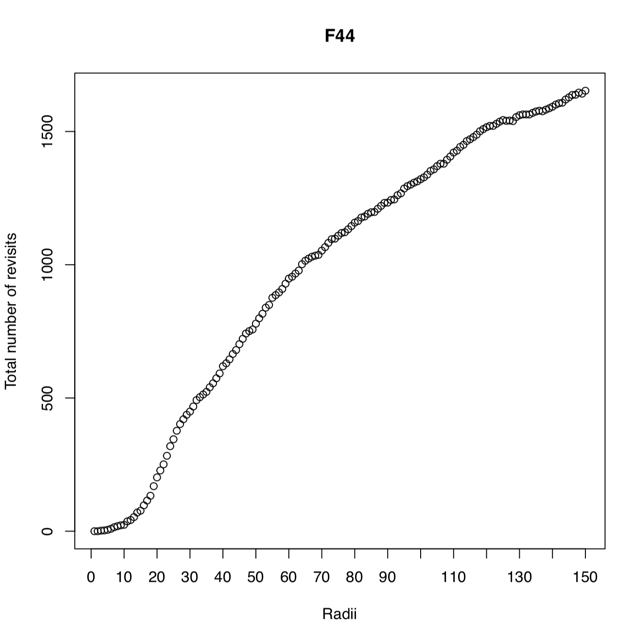

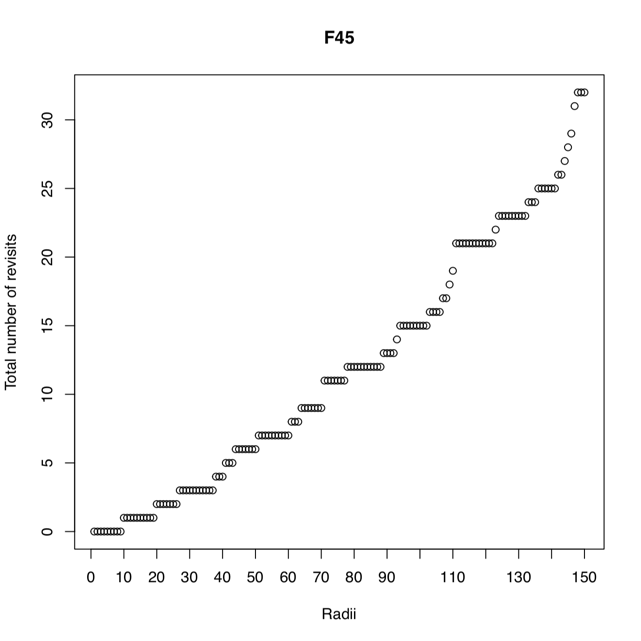

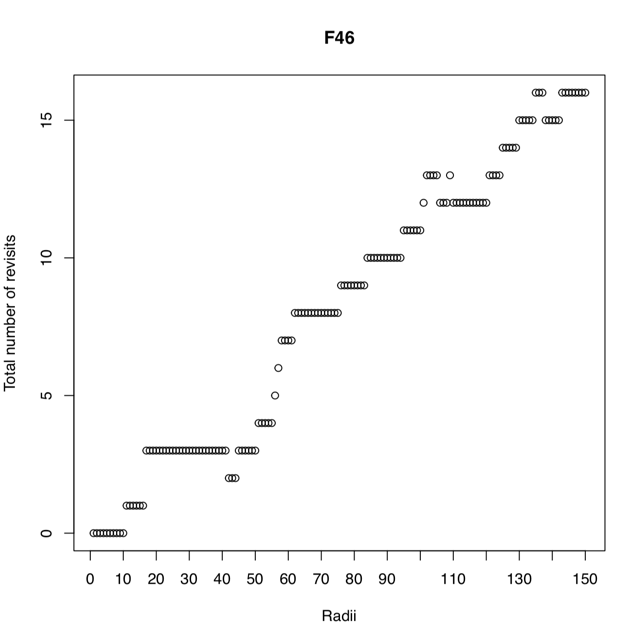

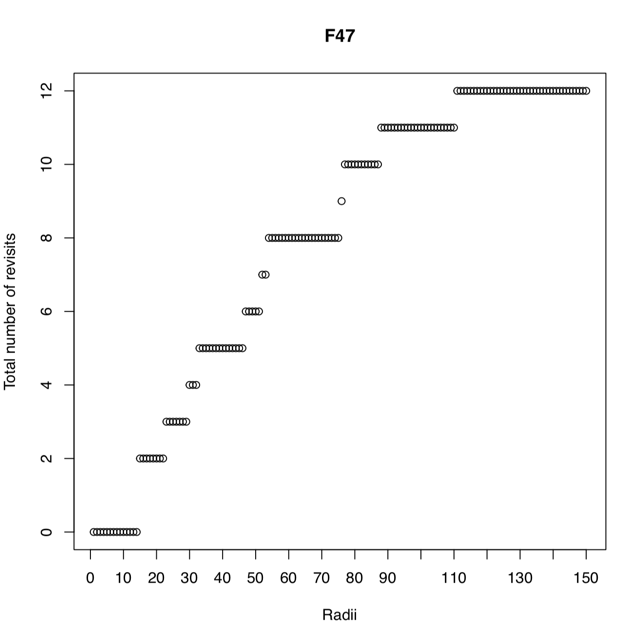
**
