# Supplementary material for: The spatial and temporal exploitation of anthropogenic food sources by common ravens (Corvus corax) in the Alps
Source: Mov Ecol. 2022 Aug 25;10:35. doi: 10.1186/s40462-022-00335-4 (PMC9414151; doi:10.1186/s40462-022-00335-4)
Supplement: Supplementary file 5 — Additional file 5. The top-ranking models for (a) the occurrence distribution, (b) average maximum daily displacement, (c) the number of anthropogenic food sources (AFSs) visited and (d) the probability of being at any AFS of raven, Corvus corax, individuals tracked in the Austrian Alps from a cut-off at ΔAICc ≤ 6 (Akaike’s Information Criterion corrected for small sample sizes). The global model for all response variables included sex, origin (i.e., wild-caught, captive-released), age class (i.e., juvenile, adult), season (i.e., autumn, winter, spring, summer) and year as fixed factors. Models (a), (b) and (c) included the ratio of the number of GPS fixes to tracking days for each individual-season combination as a fixed factor. Individual identity was included as the random factor in all models. We included an additional observation-level random effect (ORLE) in model (d) to account for overdispersion. The number of model parameters (Df), log-likelihood (logLik), AICc, ∆AICc, and model weights (ώi) are presented. [file 40462_2022_335_MOESM5_ESM.docx]

**Additional file 5** The top-ranking models for **(a)** the occurrence distribution, **(b)** average maximum daily displacement, **(c)** the number of anthropogenic food sources (AFSs) visited and **(d)** the probability of being at any AFS of raven, *Corvus corax*, individuals tracked in the Austrian Alps from a cut-off at ΔAICc ≤ 6 (Akaike’s Information Criterion corrected for small sample sizes). The global model for all response variables included sex, origin (i.e., wild-caught, captive-released), age class (i.e., juvenile, adult), season (i.e., autumn, winter, spring, summer) and year as fixed factors. Models (a), (b) and (c) included the ratio of the number of GPS fixes to tracking days for each individual-season combination as a fixed factor. Individual identity was included as the random factor in all models. We included an additional observation-level random effect (ORLE) in model (d) to account for overdispersion. The number of model parameters (Df), log-likelihood (logLik), AICc, ∆AICc, and model weights (ώ_i_) are presented.

| (a) Occurrence distribution (n = 376 estimates from 81 birds) | | | | | |
| --- | --- | --- | --- | --- | --- |
| *Top model set* | **Df** | **logLik** | **AICc** | **ΔAICc** | **ώ_i_** |
| Age class + Origin + Season + Year + Fixes by days | 12 | -690.3 | 1405.4 | 0 | 0.36 |
| Age class + Origin + Season + Year | 11 | -691.6 | 1405.8 | 0.47 | 0.29 |
| Age class + Origin + Season + Year + Sex + Fixes by days | 13 | -689.8 | 1406.7 | 1.30 | 0.19 |
| Age class + Origin + Season + Year + Sex | 12 | -691.1 | 1407.1 | 1.70 | 0.15 |
|  |  |  |  |  |  |
| (b) Average maximum daily displacement (n = 376 estimates from 81 birds) | | | | | |
| *Top model set* | **Df** | **logLik** | **AICc** | **ΔAICc** | **ώ_i_** |
| Age class + Origin + Year + Fixes by days | 9 | -449.2 | 916.9 | 0 | 0.37 |
| Age class + Origin + Season + Year + Fixes by days | 12 | -446.1 | 917.0 | 0.15 | 0.34 |
| Age class + Origin + Year + Fixes by days + Sex | 10 | -449.2 | 919.0 | 2.10 | 0.13 |
| Age class + Origin + Season + Year + Fixes by days + Sex | 13 | -446.1 | 919.2 | 2.29 | 0.12 |
| Age class + Origin + Season + Year | 11 | -449.8 | 922.4 | 5.54 | 0.02 |
|  |  |  |  |  |  |
| (c) Number of AFSs visited (n = 376 estimates from 81 birds) | | | | | |
| *Top model set* | **Df** | **logLik** | **AICc** | **ΔAICc** | **ώ_i_** |
| Age class + Origin + Season | 7 | -660.6 | 1335.4 | 0.00 | 0.34 |
| Age class + Origin + Season + Sex | 8 | -660.3 | 1337.0 | 1.58 | 0.16 |
| Age class + Origin + Season + Fixes by days | 8 | -660.4 | 1337.3 | 1.84 | 0.14 |
| Age class + Origin + Season + Year | 10 | -659.0 | 1338.6 | 3.22 | 0.07 |
| Age class + Origin + Season + Sex + Fixes by days | 9 | -660.2 | 1338.9 | 3.45 | 0.06 |
| Origin + Season | 6 | -663.4 | 1339.0 | 3.57 | 0.06 |
| Origin + Season + Sex | 7 | -663.0 | 1340.3 | 4.89 | 0.03 |
| Age class + Origin + Season + Year + Sex | 11 | -658.8 | 1340.3 | 4.90 | 0.03 |
| Age class + Origin + Season + Year + Fixes by days | 11 | -659.0 | 1340.7 | 5.26 | 0.02 |
| Origin + Season + Fixes by days | 7 | -663.3 | 1340.9 | 5.44 | 0.02 |
| Origin + Season + Year | 9 | -661.2 | 1340.9 | 5.49 | 0.02 |
|  |  |  |  |  |  |
| (d) Probability of being at any AFS (n = 369 estimates from 79 birds) | | | | | |
| *Top model set* | **Df** | **logLik** | **AICc** | **ΔAICc** | **ώ_i_** |
| Season + Year | 9 | -2381.9 | 4782.3 | 0.00 | 0.35 |
| Age class + Season + Year | 10 | -2381.4 | 4783.5 | 1.19 | 0.19 |
| Season + Year + Sex | 10 | -2381.9 | 4784.4 | 2.09 | 0.12 |
| Origin + Season + Year | 10 | -2381.9 | 4784.4 | 2.11 | 0.12 |
| Age class + Season + Year + Sex | 11 | -2381.4 | 4785.5 | 3.26 | 0.07 |
| Age class + Origin + Season + Year | 11 | -2381.4 | 4785.6 | 3.30 | 0.07 |
| Origin + Season + Year + Sex | 11 | -2381.9 | 4786.5 | 4.22 | 0.04 |
| Age class + Origin + Season + Year + Sex | 12 | -2381.4 | 4787.7 | 5.38 | 0.02 |
