# Supplementary material for: The spatial and temporal exploitation of anthropogenic food sources by common ravens (Corvus corax) in the Alps
Source: Mov Ecol. 2022 Aug 25;10:35. doi: 10.1186/s40462-022-00335-4 (PMC9414151; doi:10.1186/s40462-022-00335-4)
Supplement: Supplementary file 6 — Additional file 6. Spatial distribution of the 81 GPS tagged ravens appearing in the dataset in the Austrian Alps. The coloured polygons represent the different individuals’ 95% occurrence distributions in relation to the location of 45 anthropogenic food sources that were identified in this area, grouped by resource type. Major cities are indicated as white and black circles. The box in the upper left corner shows the field site in Austria and the countries neighbouring Austria. Thick black lines show the country borders. [file 40462_2022_335_MOESM6_ESM.docx]

**Additional file 6** Spatial distribution of the 81 GPS tagged ravens appearing in the dataset in in the Austrian Alps. The coloured polygons represent the different individuals’ 95% occurrence distributions in relation to the location of 45 anthropogenic food sources that were identified in this area, grouped by resource type. Major cities are indicated as white and black circles. The box in the upper left corner shows the field site in Austria, and the countries neighbouring Austria. Thick black lines show the country borders.
