# Supplementary material for: The spatial and temporal exploitation of anthropogenic food sources by common ravens (Corvus corax) in the Alps
Source: Mov Ecol. 2022 Aug 25;10:35. doi: 10.1186/s40462-022-00335-4 (PMC9414151; doi:10.1186/s40462-022-00335-4)
Supplement: Supplementary file 7 — Additional file 7. Model averaging outputs for the top models based on ∆AICc <= 6 for (a) the occurrence distribution (log-normal distribution), for 81 individual ravens, and (b) the probability of an individual being at any AFS (binomial error distribution) for 79 individual ravens GPS-tagged for 2.75 years in the Austrian Alps with different intercept levels for the categorical predictor ‘season’. [file 40462_2022_335_MOESM7_ESM.docx]

**Additional file 7** Model averaging outputs for the top models based on ∆AICc <= 6 for **(a)** the occurrence distribution (log-normal distribution), for 81 individual ravens, and **(b)** the probability of an individual being at any AFS (binomial error distribution) for 79 individual ravens GPS-tagged for 2.75 years in the Austrian Alps with different intercept levels for the categorical predictor ‘season’.

**Table 1**

| 1. Occurrence distribution: Log-transformed model estimates | | | | |
| --- | --- | --- | --- | --- |
| **Fixed effects** | **Estimate** | **Unc. SE** | **95% CI** | **RVI** |
| *Intercept* | -1.02 | 0.73 | [-2.45,0.42] |  |
| Juvenile^Ϯ^ | -0.84 | 0.25 | **[-1.34, -0.34]** | **1.00** |
| Wild-caught^+^ | 2.20 | 0.43 | **[1.36, 3.04]** | **1.00** |
| Autumn^‡^ | 0.44 | 0.25 | **[-0.05, 0.93]** | **1.00** |
| Spring^‡^ | -2.30 | 0.28 | **[-2.85, -1.74]** | **"** |
| Summer^‡^ | -1.34 | 0.25 | **[-0.84, -0.85]** | **"** |
| 2018^*^ | 1.59 | 0.44 | **[0.73, 2.45]** | **1.00** |
| 2019^*^ | 2.03 | 0.48 | **[1.08, 2.98]** | **"** |
| 2020^*^ | 3.64 | 0.61 | **[2.44, 4.84]** | **"** |
| Male^ǂ^ | 0.37 | 0.29 | [-0.40, 1.14] | 0.35 |
| Fixes by days | -0.30 | 0.21 | [-0.68, 0.07] | 0.55 |
| 1. Number of AFSs visited: Log-transformed model estimate | | | | |
| **Fixed effects** | **Estimate** | **Unc. SE** | **95% CI** | **RVI** |
| *Intercept* | 0.78 | 0.22 | [0.34, 1.22] |  |
| Juvenile^Ϯ^ | -0.21 | 0.11 | **[-0.38, -0.03]** | **0.86** |
| Wild-caught^+^ | 0.48 | 0.12 | **[0.24, 0.72]** | **1.00** |
| Autumn^‡^ | -0.35 | 0.09 | **[-0.52, -0.18]** | **1.00** |
| Spring^‡^ | -0.36 | 0.11 | **[-0.57, -0.14]** | **"** |
| Summer^‡^ | -0.39 | 0.10 | **[-0.58, -0.20]** | **"** |
| 2018^*^ | 0.34 | 0.17 | [-0.22, 0.91] | 0.15 |
| 2019^*^ | 0.40 | 0.18 | [-0.18, 0.98] | " |
| 2020^*^ | 0.31 | 0.17 | [-0.34, 0.96] | " |
| Male^ǂ^ | 0.07 | 0.06 | [-0.13, 0.28] | 0.29 |
| Fixes by days | -0.04 | 0.05 | [-0.22, 0.14] | 0.26 |
| 1. Probability of being at any AFS: Logit-transformed model estimates | | | | |
| **Fixed effects** | **Estimate** | **Unc. SE** | **95% CI** | **RVI** |
| *Intercept* | -1.83 | 0.34 | [-2.49, -1.16] |  |
| Juvenile^Ϯ^ | -0.13 | 0.10 | [-0.40, 0.14] | 0.36 |
| Wild-caught^+^ | 0.00 | 0.10 | [0.39, 0.39] | 0.26 |
| Autumn^‡^ | -0.60 | 0.14 | **[-0.87, -0.32]** | **1.00** |
| Spring^‡^ | 0.57 | 0.15 | **[0.27, 0.86]** | **"** |
| Summer^‡^ | 0.07 | 0.14 | **[-0.21, 0.35]** | **"** |
| 2018^*^ | 0.58 | 0.26 | **[0.07, 1.09]** | **1.00** |
| 2019^*^ | -0.04 | 0.28 | **[-0.58, 0.50]** | **"** |
| 2020^*^ | -0.09 | 0.34 | **[-0.75, 0.57]** | **"** |
| Male^ǂ^ | -0.04 | 0.09 | [-0.39, 0.31] | 0.26 |

Reference categories: ϮAge class: ‘adult’, +origin: ‘captive-released’, ‡season: ‘**winter**’, *year: ‘2017’, and ǂsex: ‘female’

**Table 2**

| 1. Occurrence distribution: Log-transformed model estimates | | | | |
| --- | --- | --- | --- | --- |
| **Fixed effects** | **Estimate** | **Unc. SE** | **95% CI** | **RVI** |
| *Intercept* | -2.36 | 0.64 | [-3.62, -1.10] |  |
| Juvenile^Ϯ^ | -0.84 | 0.25 | **[-1.34, -0.34]** | **1.00** |
| Wild-caught^+^ | 2.20 | 0.43 | **[1.36, 3.04]** | **1.00** |
| Autumn^‡^ | 1.78 | 0.18 | **[1.42, 2.14]** | **1.00** |
| Spring^‡^ | -0.95 | 0.22 | **[-1.39, -0.51]** | **"** |
| Winter^‡^ | 1.34 | 0.25 | **[0.85, 1.84]** | **"** |
| 2018^*^ | 1.59 | 0.44 | **[0.73, 2.45]** | **1.00** |
| 2019^*^ | 2.03 | 0.48 | **[1.08, 2.98]** | **"** |
| 2020^*^ | 3.64 | 0.61 | **[2.44, 4.84]** | **"** |
| Male^ǂ^ | 0.37 | 0.29 | [-0.40, 1.14] | 0.35 |
| Fixes by days | -0.30 | 0.21 | [-0.68, 0.07] | 0.55 |
| 1. Probability of being at any AFS: Logit-transformed model estimates | | | | |
| **Fixed effects** | **Estimate** | **Unc. SE** | **95% CI** | **RVI** |
| *Intercept* | -1.76 | 0.30 | [-2.35, -1.16] |  |
| Juvenile^Ϯ^ | 0.13 | 0.10 | [-0.40, 0.14] | 0.36 |
| Wild-caught^+^ | 0.00 | 0.10 | [0.39, 0.39] | 0.26 |
| Autumn^‡^ | -0.67 | 0.11 | **[0.88, -0.45]** | **1.00** |
| Spring^‡^ | 0.49 | 0.12 | **[0.25, 0.74]** | **"** |
| Winter^‡^ | -0.07 | 0.14 | **[-0.35, 0.21]** | **"** |
| 2018^*^ | 0.58 | 0.26 | **[0.07, 1.09]** | **1.00** |
| 2019^*^ | -0.04 | 0.28 | **[-0.58, 0.50]** | **"** |
| 2020^*^ | -0.09 | 0.34 | **[-0.75, 0.57]** | **"** |
| Male^ǂ^ | -0.04 | 0.09 | [-0.39, 0.31] | 0.26 |

Reference categories: ϮAge class: ‘adult’, +origin: ‘captive-released’, ‡season: ‘**summer**’, *year: ‘2017’, and ǂsex: ‘female’
